# A model for chromosome organization during the cell cycle in live *E. coli*

**DOI:** 10.1038/srep17133

**Published:** 2015-11-24

**Authors:** Yuru Liu, Ping Xie, Pengye Wang, Ming Li, Hui Li, Wei Li, Shuoxing Dou

**Affiliations:** 1 Key Laboratory of Soft Matter Physics, Beijing National Laboratory for Condensed Matter Physics, Institute of Physics, Chinese Academy of Sciences, Beijing 100190, China

## Abstract

Bacterial chromosomal DNA is a highly compact nucleoid. The organization of this nucleoid is poorly understood due to limitations in the methods used to monitor the complexities of DNA organization in live bacteria. Here, we report that circular plasmid DNA is auto-packaged into a uniform dual-toroidal-spool conformation in response to mechanical stress stemming from sharp bending and un-winding by atomic force microscopic analysis. The mechanism underlying this phenomenon was deduced with basic physical principles to explain the auto-packaging behaviour of circular DNA. Based on our observations and previous studies, we propose a dynamic model of how chromosomal DNA in *E. coli* may be organized during a cell division cycle. Next, we test the model by monitoring the development of HNS clusters in live *E. coli* during a cell cycle. The results were in close agreement with the model. Furthermore, the model accommodates a majority of the thus-far-discovered remarkable features of nucleoids *in vivo*.

The bacterium is considered a simple life form. Among bacteria, *E. coli* is the most studied organism as a model system via developed molecular manipulation methods of genes and proteins in live cells. Scientists are attempting to learn more about the mechanisms of maintaining a living organism from *E. coli* with the goal of understanding how gene transcription is regulated and how a cell reacts to external and internal stimuli^1^,^2^. Despite being a simple life form, unexpected patterns of chromosomal DNA compaction and organization within these tiny organisms have been observed^2^,^3^. In live *E. coli* cells supplied with required nutrients, chromosomal DNA is simultaneously amplified and compacted to fit into the confined cytoplasmic volume. *E. coli* precisely passage their chromosomal DNA and proteins through generations in a smooth and elegant manner, underlying an efficient and reproducible large-scale organization^1^,^4^.

In bacteria, abundant nucleoid-associated proteins (NAPs) are associated with chromosomes to facilitate the compaction of chromosomal DNA^5^,^6^. In *E. coli*, major NAPs include HNS, HU, Fis, IHF, and StpA^7^. Each of these NAPs non-specifically binds to DNA and is involved in gene regulation and chromosome compaction^8^,^9^,^10^,^11^. In addition to NAPs, entropy stemming from molecular crowding in a confined intracellular environment contributes to the process of DNA compaction^12^,^13^. Theoretical analysis has provided evidence that DNA compaction occurs under the influence of a subtle equilibrium between several competing factors^14^. With the aid of chromosomal locus labelling and live cell imaging, the amplification and segregation dynamics of chromosomes in live *E. coli* have been revealed, providing insight into chromosome organization *in vivo*^5^. Four macrodomains (MDs) and two less-structured MDs inside the nucleoid have been discovered using homologous recombination rate analysis and fluorescence in situ hybridization (FISH) staining, revealing higher order structural domains in the *E. coli* chromosome^15^,^16^. Remarkable features of nucleoids were recently discovered via high spatial and temporal resolution microscopy of live *E. coli* cells. Two clusters of HNS per copy of *E. coli* chromosomal DNA were identified by Wang *et al.*, indicating that higher order organization of chromosomal DNA is likely^7^. Helical ellipsoid nucleoids with two bundles confined by the radial cell periphery were visualized by 4D microscopic monitoring of fluorescence-tagged HU proteins, revealing striking nucleoid dynamics during the cell division cycle^17^. Overall, the organization of chromosomal DNA in live bacteria is quite different from that of eukaryotes (the ‘beads on a string’ conformation) and viral DNA (confined in a tightly compacted capsid). The nucleoid of bacteria is highly compacted but with soft nature to be dynamic accessible to motor proteins^18^,^19^.

## Results

### Auto-packaging behaviour of plasmid DNA

**Figure 1:**
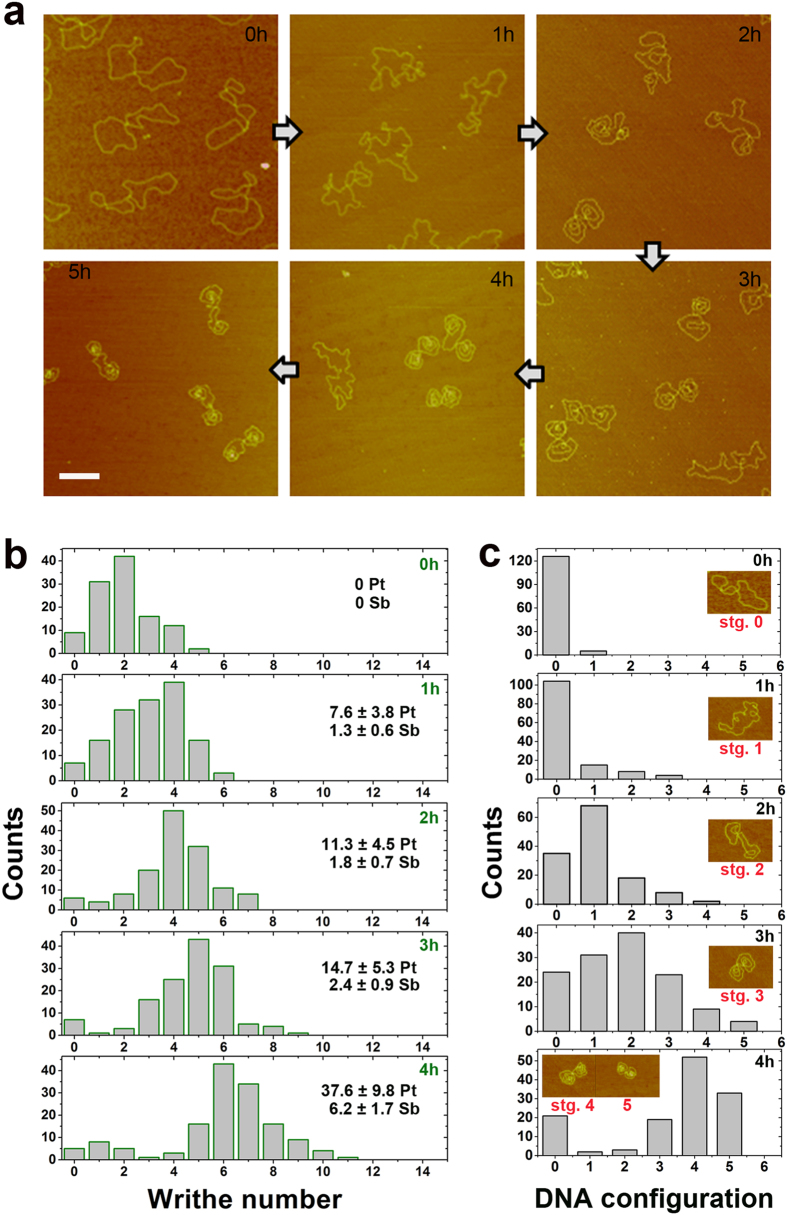
Auto-packaging of plasmid DNA in response to mechanical stress of C and T co-ordination. (**a**) AFM visualization of plasmid pBR322 DNA molecules gradually packaged into an eight-shaped dual-toroidal-spool conformation. The DNA molecules were incubated with a C and T mixture for ~5 h. Scale bar is 200 nm. (**b**) Statistical analysis of writhe number per pBR322 DNA molecule in the samples from (**a**) collected at different incubation times. The amount of divalent bound platinum (Pt) and sharp bends (Sb) per DNA molecule are indicated as the mean ± SD. (**c**) Distribution of DNA molecule compositions with different conformations during incubation corresponding to (**b**) under our experimental conditions. The number on horizontal axis refers to stage 0–5. Inset, representative structure of DNA molecules at each stage.

In our previous study of the effect of platinum drugs on single DNA molecules, the unexpected phenomenon of circular plasmid DNA auto-wrapping in response to transplatin (T) and cisplatin (C) co-ordination was observed^20^. We next investigated the dynamics and process of plasmid DNA auto-packaging (Fig. 1). Surprisingly, pBR322 DNA molecules froze into a symmetric dual-toroidal-spool conformation after incubation with C and T for several hours, as observed with atomic force microscopic (AFM) analysis. C and T co-ordination (C+T) introduced sharp bends of up to 180° and resulted in the untwisting of local DNA fragments^20^. However, pBR322 DNA incubated with the same molar concentration of C or T alone did not exhibit this auto-packaging behaviour; instead, it mainly formed local micro-loops or bends during the incubation. Given the comparable amount of divalent Pt atoms detected in C and C+T samples by inductively coupled plasma mass spectrometry (ICP-MS), insufficient mechanical stress injected by C alone compared with C+T might underlie the marked difference in DNA conformation between the two groups^20^. When negative supercoiled plasmid DNA was injected with ~1.3 sharp bends by ~7.6 ± 3.8 (mean ± SD, *n* = 3) divalent crosslinked Pt atoms per DNA molecule, plasmid DNA began to alter its conformation, and the average writhe number increased to ~3.0 ± 0.1 (mean ± s.e.m., *N* = 141; 1 h, Fig. 1a,b; intact DNA mean writhe number ~1.9 ± 0.1, mean ± s.e.m., *N* = 112). Meanwhile, the writhe joints moved from the centre region (0 h) to the periphery (1 h) of the DNA molecules, indicating increased tension inside the DNA molecules^21^ (Supplementary Fig. S1). With the sharp bends increased to ~1.8–2.4 bearing ~11.3–14.7 divalent Pt atoms per DNA molecule, the DNA molecules exhibited writhe numbers as high as ~4.2–5.1 (2 ~ 3 h, Fig. 1a,b). Gradually, the DNA molecules settled into an eight-shaped dual-toroidal-spool conformation and became more tightly compacted, with an average writhe number ~6.1±0.2 (mean±s.e.m., *N* = 145), during which time divalently linked Pt atoms accumulated up to ~37.6 per DNA molecule (4 h, Fig. 1a,b). The number of sharp bends was calculated as 16 percent of the total Pt atoms divalently crosslinked to DNA, as determined by a combination of ICP-MS and denaturing gel electrophoresis^20^. The calculated number of sharp bends, and the average increased writhe number counted by AFM (Supplementary Fig. S2) performed on the same DNA sample during the 1–4 h incubation, were coincidently close to each other (Fig. 1b). The highest DNA density was observed in the centre core of the spools by AFM (Supplementary Fig. S1). The rearrangement of intact plasmid DNA into a mature dual-toroidal-spool conformation occurred in five stages, as shown in Fig. 1c and Supplementary Fig. S1. DNA molecules in the dual-toroidal-spool conformation exhibited a symmetric distribution of two parts of DNA content. In each of the spools, DNA was wrapped onto itself, with the ends crossing with each other and looping into the remaining spool.

### Mechanism of DNA auto-packaging

By the end of our experiments, over 90% of the DNA molecules had uniformly frozen into the dual-toroidal-spool conformation upon sharp bending and unwinding stress by C+T. The remaining ~10% of DNA molecules were nicked and retained an open circular conformation. These results demonstrate that the dual-toroidal-spool conformation represents the energetically lowest mechanical state and predominates among all the other possible conformations for circular DNA to compensate for sharp bending and untwisting (C+T) mechanical stress in bulk. We next deduced the basic physical principles underlying the predominant conformation. We first analysed the local mechanical effect of external bending factors on a normal B-form DNA fragment (Supplementary Fig. S3). Bending factors exert action *F*, which is generally perpendicular to the DNA strand and points to bending factors. Thus, DNA will generate a reaction *F*′ that is opposite to *F*, according to Newton’s third law. Bended DNA strands generate *F*′ via their intrinsic tendency to return back to the B-form conformation due to the elastic property of helical DNA. Local deformation of DNA strands introduces uneven distributed tension along the DNA backbone. The extra torque mechanical stress continues to propagate along the circular DNA backbone, which cannot be released from the ends as in the case of linear DNA. Macromolecules such as DNA and proteins fluctuate into a lower energetically mechanical state. When the DNA backbone is nearly spherical or ellipsoidal, the deformation of DNA is most evenly distributed across the whole DNA molecule. DNA might then freeze into this configuration (Supplementary Fig. S4a). In the dual-spool conformation, tension along the DNA backbone that is generated inside one spool by the effects of DNA coiling and bending propagates to the entering and exiting ends, where it turns to serve as the supporting force that acts on the resting spool, thereby holding the coiled DNA strands together to prevent them from elastically spreading into B-form DNA (Supplementary Fig. S4b). Meanwhile, the symmetric conformation of the dual-toroidal-spool achieves a self-balanced structure.

**Figure 2:**
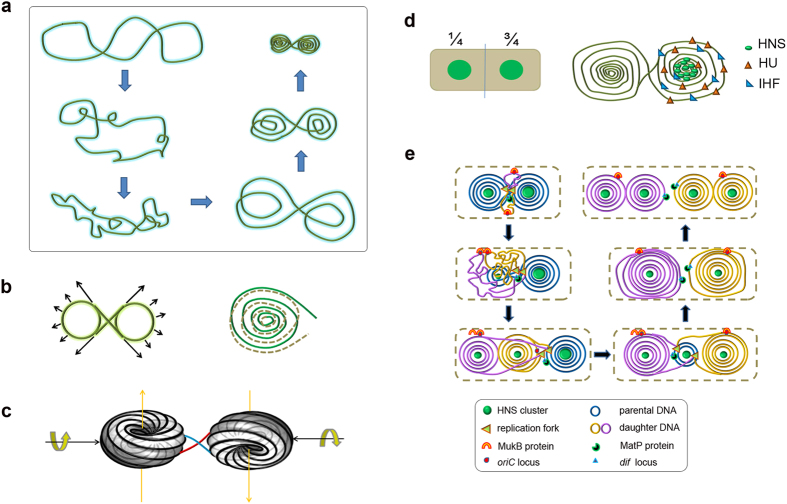
Proposed model for chromosome organization in *E. coli*. (**a**) Behaviour of circular plasmid DNA auto-packaging upon sharp bending and unwinding mechanical stress in bulk, as illustrated in a 2D diagram deduced from AFM analysis. (**b**) Eight-shaped scaffold (green) for the dual-toroidal-spool conformation (left panel). Black arrows indicate tension along the DNA backbone (long arrows) and elastic anti-bending reactions (*F*′) generated in DNA strands (short arrows). In each spool, DNA wrapped onto itself to form numerous DNA circles (right panel). One half of each DNA circle signifies the entry of DNA strands (green solid lines), while the other half signifies the exit of strands (grey dashed lines). (**c**) Proposed 3D model of chromosome organization in *E. coli.* A rotational dual-toroidal-spool (black, adapted from reference 26) with one spool upside down relative to the other was linked by a buckled hinge (blue and red). The hinge region is widened in the image for clarity (see details in Supplementary Figs S7, 8). Each spool retained a counter-clockwise rotational tendency along the long axis relative to the hinge. (**d**) NAP distribution in nucleoids. Left panel, positions for HNS clusters, as demonstrated previously^7^. Right panel, only a portion of DNA strands and NAPs were drawn for clarity, illustrating the general dispersed distribution of HU and IHF, and a clustered HNS distribution among nucleoids. (**e**) Proposed model of dynamic chromosome organization in *E. coli* during a cell division cycle. DNA strands containing *oriC* chromosomal loci were pulled from one spool (blue, upper-left panel) to proceed through the replication fork, thereby initiating DNA replication. The newly synthesized DNA collapsed into two new docked spools (purple and yellow, left-bottom panel) during the middle stage of DNA replication. During the late stage of DNA replication, one of the newly docked spools across the remaining parental spool (blue) was disposed at the other half of the cell (right-bottom panel). The docked spools were isolated from replication machinery after the completion of DNA replication and were re-arranged into a dual-toroidal-spool conformation in the ready-to-divide cells (right-upper panel).

The DNA strands are loosely coiled during early stages (0–1 h) and then become more tightly coiled together in each dual-toroidal-spool with increasing injection of C+T mechanical stress (2–4 h Figs 1a and 2a). In turn, writhe formation compensates for untwisting mechanical stress, such that the spools are increasingly compacted to accommodate the generated writhes (i.e., new DNA circles). The DNA bending energy increases from the periphery (~0.91 k_B_T per DNA circle) to the centre core (~5.44 k_B_T per DNA circle) due to the aggravated deformation and shortened radius of DNA circles in the centre of the spool relative to the periphery (2–4 h Fig. 1a and Supplementary Fig. S5).

Dual symmetry was the only observed form of symmetry in the auto-packaged plasmid DNA. Neither triplex symmetry nor quadruplex symmetry were observed. While this is an interesting phenomenon, we cannot address it in the present manuscript. We were only able to calculate the relative probability of dual-spool and multi-spool occurrence inside a circular DNA molecule. The results of this calculation indicated the occurrence of a triplex-spool with *N*^*−3*^ probability (*N* = all possible distributions of transient forces along a DNA fragment during a period of time) or a quadruplex-spool with *N*^*−4*^ probability to attain mechanically balanced states that allow DNA to freeze, compared with a dual-spool of *N*^*−2*^ probability, in the case of several comparable local toroidal spools formed spontaneously in circular DNA (Supplementary Fig. S6). More practically, a multiple-spool comprised of one circular DNA molecule had an energetically higher mechanical state that fluctuated into a lower mechanical state, such as dual-spool or single-spool. For example, one pBR322 DNA molecule at a length of 4,361 bps compacted into a single-spool with one DNA circle has a total bending energy ~0.695 k_B_T; if it compacts into dual-spool with one DNA circle in each spool, the total bending energy is ~2.778 k_B_T, and ~6.255 k_B_T of the total bending energy for it in triplex-spool with one DNA circle in each spool. A single-spool is an attractive conformation for a circular DNA to freeze into, and it resides in a lower energetic state than the dual-spool. However, circular DNA coiled in one spool configuration could not effectively achieve a self-balanced state (i.e., the coiled spherical DNA strands were not effectively held together by supporting tension). Therefore, the coiled DNA strands underwent outward fluctuation and were constrained by the circular DNA itself. The strands finally settled in a dual-spool self-balanced structure (Supplementary Fig. S9b,c).

### Model for *E. coli* chromosome organization

Plasmid DNA and bacterial chromosomal DNA are similar in that they are negative supercoiled circular DNA of differing sizes. The mechanical effect of C+T co-ordination to IHF and HU proteins in the local DNA backbone is analogous (i.e., both introduce sharp bends and un-winding of DNA)^20^,^22^,^23^. These analogies intrigue us to speculate on the organization pattern of chromosomal DNA in *E. coli*. It is notable that the phenomenon from plasmid DNA are not always feasible for chromosomal DNA. Prokaryotic chromosomal DNA has a molecular weight thousands of times that of plasmid DNA, and thus exhibits large topological domains as well as more complicated compaction pathways. Nevertheless, the underlying physical principles might be universal and applicable to different molecules under similar conditions. Thus, we propose a hypothesis that the chromosomal DNA of *E. coli* is organized into a dual-toroidal-spool conformation (upper right panel in Fig. 2a). The nucleoid had an eight-shaped scaffold with DNA coiled onto itself in each spool to form a buckled balanced dual-toroidal-spool conformation (Fig. 2b). In this model, the centre core of each spool corresponds to the HNS cluster observed by photoactivated localization microscopy (PALM) *in vivo*^7^. The dual-toroidal-spool conformation well accommodates the observation that one copy of chromosomal DNA bearing two HNS clusters symmetrically positioned at cell quarters. Given that the highest DNA density is detected in the centre core, adjacent DNA strands fall within close proximity of each other, so HNS is spatially accessible and can bridge them because force and proximity of the DNA duplex are important for HNS bridging as revealed by single molecular investigations^19^,^24^,^25^. In this model, HNS serves as spacer chaperone to protect DNA from collision (Fig. 2d). Other NAPs (e.g., HU, IHF and Fis) function as bending and unwinding chaperones that inject mechanical stress and accelerate the compaction of chromosomal DNA. Mechanical stress injection enhances tension inside DNA molecules. When certain amounts of HU, IHF and Fis are bound to DNA, compaction plateaus and the tension in DNA strands becomes so high that further binding of NAPs is unfavourable. Thus, compaction is equilibrated and stabilized.

Based on our AFM analysis and other theoretical studies, we propose a 3D model for nucleoid organization as a rotational dual-toroidal-spool (Fig. 2c)^26^. Each spool shares the same essential configuration because they are developed under similar mechanical stress by the same NAPs. One spool faces to one side, while the other spool is approximately upside down relative to the first (Supplementary Fig. S7). The entering and exiting ends of each spool buckle together and serve as a hinge for the nucleoid. As eight-shaped negative DNA supercoils, each spool exhibits a counter-clockwise 3D rotational tendency along the long axis of the nucleoid relative to the hinge (Supplementary Fig. S8a–c). The two spools rotate relative to the hinge in the middle, and are buckled together to obtain a balanced 3D structure. The orbit of the DNA strands in each spool projected on the main plane is similar to the 2D model, as shown in Fig. 2a and Supplementary Fig. S8d. To compensate for the bending tension, the DNA strands in a spool rotate around the orbit to form a rotational toroidal spool ellipsoid in 3 dimensions, as elucidated in a previous theoretical study^26^.

Based on our observations and other related work, such as the findings that the replication factory is located at the centre of the *E. coli* cell, that the DNA strands were pulled through the replication machinery during DNA replication^27^,^28^ and that the *oriC* and *dif* loci have distinct cytoplasmic locations^16^,^29^,^30^, we further propose a dynamic model for chromosome organization during a cell division cycle in live *E. coli* (Fig. 2e). Firstly, the replication factory located at the centre of *E. coli* cells pulls the DNA strands from one spool containing the *oriC* chromosomal loci, thereby initiating replication. The newly synthesised DNA strands are dispersed randomly in the cytoplasm, whereas the chromosomal loci in the Ori region are docked on cellular counterparts near cell quarters by MukB proteins and the *migS* cis element^31^,^32^. Upon the binding of NAPs to newly replicated DNA, the actual torsional constrained newborn DNA^33^,^34^, collapses into a single-spool docked on replication machinery during DNA replication (Supplementary Fig. S9). Each sister DNA forms one spool. DNA is continually pulled from the un-replicated parental DNA spool. Newborn DNA strands undergo free diffusion and stress from NAPs binding, and they are wrapped onto the docked sister spools (Fig. 2e). Finally, the docked spool is isolated from the replication machinery when DNA replication is completed, and DNA is released from DNA polymerases. Once released, the single-spool, which contains one copy of sister chromosomal DNA, rearranges into the dual-toroidal-spool conformation to obtain a balanced configuration (Supplementary Fig. S9 b,c).

### HNS cluster development in live *E. coli*

To test our hypotheses, we directly monitored HNS cluster development during a cell cycle in *E. coli* using PALM. Moreover, we checked the model with published data related to nucleoid behaviour and structural features *in vivo* to analyse the accommodation ability of the model with the experimental data. Details are available in the Supplementary Discussion.

**Figure 3:**
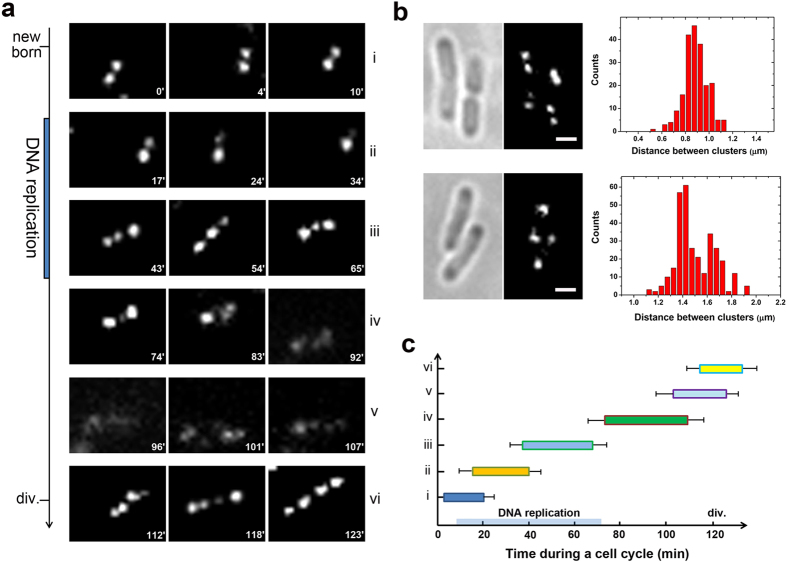
HNS cluster development visualized by PALM during a cell division cycle in live *E. coli*. (**a**) Typical HNS cluster phases during one cell cycle. Two HNS clusters at a defined distance in newborn cells are shown in (i). After DNA replication, one of the HNS clusters shrank and finally disappeared (ii). Two new clusters, which were smaller compared with the parental HNS clusters, appeared during the middle stage of DNA replication (left, iii). The size of the parental cluster decreased with increasing size of the new clusters (middle, iii). Two large HNS clusters were deposited at the two halves of the cell with the shrinking parental HNS cluster in the middle (right, iii). The middle parental HNS cluster disappeared, leaving two comparable HNS clusters in each half of the cell (left, iv). The HNS clusters subsequently became smeared (middle and right, iv). The HNS clusters remained less visible for a period of time (v). Finally, four new HNS clusters appeared in the pre-division cells (vi). The snapshots-taken time points (min) were labelled on each image, corresponding to the normalized cell cycle of ~120 min. (**b**) The distribution of distance between HNS cluster pairs (*N* = 211) in newborn and early growing cells with a cell length ~1.7-2.5 μm (upper panel) and that (*N* = 310) in middle-aged and old cells with a cell length ~2.6-3.7 μm (lower panel). (**c**) Duration of HNS clusters in phases (i–vi) within the cell cycle. The average time of a cell cycle is ~127 ± 25 (mean ± SD, *N* = 706) min, during which ~15% cells stop growing, reflecting the diversity of cellular repair ability from 405 nm phototoxicity. The different lengths of individual cell cycles were normalized to 120 min, and the corresponding time points were aligned. Error bars indicate s.e.m. Scale bars are 1 μm.

In our model, HNS clusters correspond to the centre core of DNA spools. A packaged DNA spool bore a clear HNS cluster. We tagged HNS protein with a monomeric photoactivatable fluorescent protein, mEos2, as previously described^7^. The fusion proteins were expressed from their native promoters at the chromosomal loci to obtain an expression approximately at the wild-type level. Cells were imaged in M9 minimal medium at room temperature during the early log phase. Time-lapse snapshots at 10–20 min intervals were taken during the cell division cycle, and the images were analysed sequentially. Representative conformations of HNS clusters at different stages of the replication cycle are shown in Fig. 3. We observed two, three and four typical HNS clusters in newborn, growing and ready-to-divide cells, respectively; these findings were consistent with previous observations^7^. Furthermore, the dynamic developments of two to three clusters and three to four clusters were clearly visualized by monitoring the same cell during a cell cycle, which strongly supports our model of chromosome organization in live *E. coli*. In brief, one of the two HNS clusters with an average distance of ~0.88 ± 0.1 μm (mean ± SD, *N* = 211; Fig. 3b, upper panel) in newborn cells shrank significantly, whereas the other cluster remained essentially constant (ii, Fig. 3a). The shrinking cluster disappeared around 20–30 min after DNA replication and two new additional comparably smaller HNS clusters showed up at the same half of the cells around ~30–40 min during DNA replication (iii Fig. 3a). The two new clusters grew bigger, whereas the other parental cluster shrank (iii, Fig. 3a). At the late stage of DNA replication, the new HNS cluster in the middle switched locations with the shrinking parental cluster (iii, Fig. 3a). Finally, the parental cluster disappeared and two big clusters remained in a symmetric distribution in the growing cells. The average distance between was ~1.50 ± 0.15 μm (mean ± SD, *N* = 310), with two peaks centered around 1.41 μm and 1.65 μm (Fig. 3b, lower panel). The two big clusters smeared and disappeared around 60–80 min in a cell cycle (iv–v, Fig. 3a), followed by the appearance of four new clusters around the end of a cells cycle (vi, Fig. 3a). These observations are in agreement with our proposed model. Once the docked spool was released from the replication fork, it re-arranged itself into a dual-toroidal-spool conformation to obtain a stabilized and balanced structure for circular DNA to be frozen into.

The duration times of each phase were recorded, as shown in Fig. 3c, for ~700 *E. coli* cell division cycles. The average time of a cell cycle is ~127 ± 25 min (mean ± SD, *N* = 706). The overlapping duration of adjacent phases was observed due to the heterogeneity of cell recovery ability from 405 nm irradiation. There are two phases in which two HNS clusters appeared in one *E. coli* cell. One is the newborn and early stage of DNA replication (0–20 min during a cell cycle, Fig. 3c); the other is during the late stage of DNA replication and after DNA replication (60–100 min during a cell cycle, Fig. 3c). Statistical measurements of the distance between centres of HNS cluster pairs showed a significant difference between the two phases (Fig. 3b). The distance between HNS cluster pairs in newborn and early growing cells was ~0.88 ± 0.1 μm (mean ± SD, *N* = 211) with a narrow distribution profile that was constant regardless of cell length ranged from 1.7 μm to 2.5 μm, reflecting a relatively solid structure of HNS cluster pairs in these cells. The average distance of ~1.50 ± 0.15 μm (mean ± SD, *N* = 310) between HNS cluster pairs has been observed in middle-aged and old cells and shows a broad distribution. Meanwhile, the distance values varied significantly, with the cell body length ranging from 2.6 μm to 3.7 μm, probably reflecting the isolated state of sister nucleoids at this time span. Two peaks centred around 1.41 μm and 1.65 μm were observed in middle-aged and old cells, respectively. The following scenarios might explain this observation. Before DNA replication is completed, when two sister DNAs are linked via replication forks, the 1.41 μm peak value might reflect the average distribution of HNS cluster pairs at this stage. After DNA replication is completed, sister DNA is released from the replication fork and thus the distances between might contribute to the 1.65 μm peak distribution, which is consistent with previous observations that sister DNAs move freely and fit into each cell half after dissociation from replication machinery^17^.

**Figure 4:**
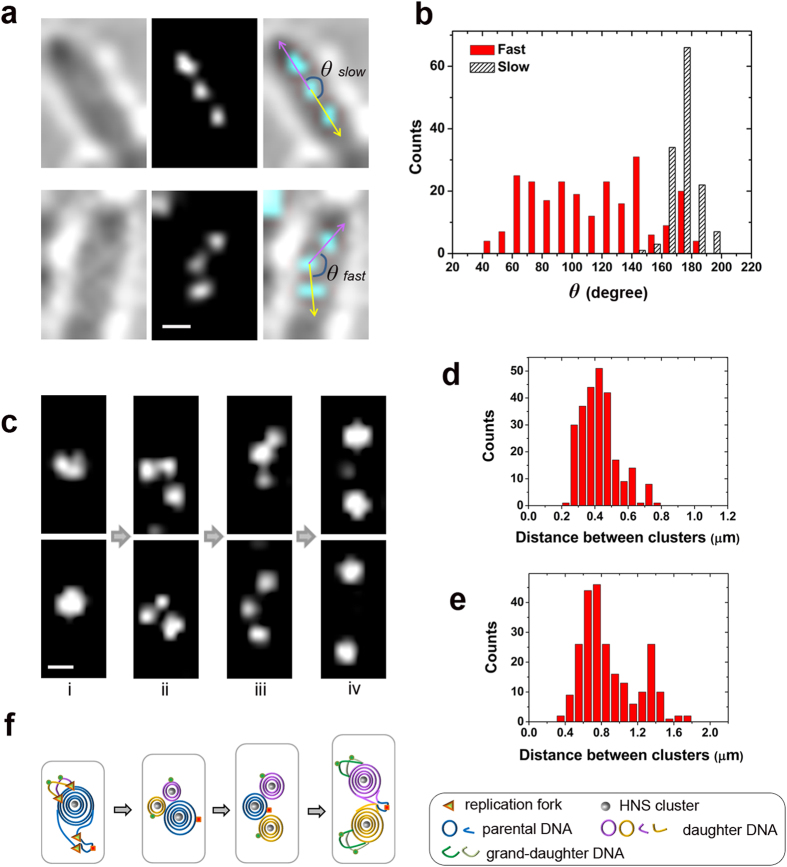
HNS cluster development pattern in a multifork *E. coli* chromosome. **(a**) The distribution pattern of three HNS clusters in cells under slow growing conditions (M9 minimal media) as shown in upper panel, is different from that of cells under fast growing conditions (LB-agarose pad, lower panel). (**b**) The distribution of angles *θ* as indicated in (**a**) under fast (*N* = 238) or slow (*N* = 133) growing conditions. (**c**) HNS clusters in a multifork chromosome developed through a ‘one-three-two’ mode over a cell cycle. The representative phase of HNS clusters in *E. coli* cells growing under an LB-agarose pad is shown as i to iv for an amplification cycle. i, pre-division cells; ii, newborn cells; iii, growing cells; iv, pre-division cells. (**d**) Distribution of distance between adjacent HNS clusters (*N* = 255) in newborn and early growing cells under an LB-agarose pad. (**e**) Distribution of distance between adjacent HNS clusters (*N* = 239) in late growth and pre-division cells growing under LB-agarose pad. (**f**) Proposed model for the organization pattern of multifork chromosomal DNA corresponding to (**c**). Scale bars are 500 nm.

HNS cluster development was also investigated using the multifork chromosome of *E. coli* cells cultured under an LB-agarose pad (Fig. 4). This experiment revealed symmetrically outward binary growth and division of the parental chromosome into two sister nucleoids, which was consistent with previous investigations^35^,^36^. The number of HNS clusters in fast growing cells was typically one, three or two during a cell cycle (Fig. 4c). The HNS clusters were found to be essentially aligned under slow growing conditions, and the angles between them were centred around ~180 degree (Fig. 4a,b). However, the three HNS clusters under fast growing conditions had a triangular distribution pattern, with the angles between them ranging from ~40 to 180 degrees (Fig. 4a,b). This observation indicates that the two sister DNA masses are not linear but rather bilobed, which is consistent with previous morphological observations of nucleoids under LB-pads^35^. The distance between adjacent HNS clusters is ~0.43 ± 0.11 μm (mean ± SD, *N* = 255) in new-born cells (Fig. 4d), which is significantly shorter than that observed in slow growing cells (~0.88 μm). The HNS cluster distance in middle aged and old cells averaged ~0.89 ± 0.30 μm (mean ± SD, *N* = 239), with one peak centred at approximately 0.7 μm for adjacent clusters and another peak centred at approximately 1.37 μm for distal clusters (Fig. 4e). These observations agree with our model that chromosomal DNA is organized into a single-spool docking on replication forks prior to the completion of DNA replication.

## Discussion

The fundamental mechanism underlying the precise and regular auto-organization of very long chromosomal DNA in a living cell remains unknown. Extensive theoretical and experimental studies have reported that NAPs in bacteria, or histones in mammal cells, play an important role in initiating the wrapping of DNA through electrostatic interactions with its phosphate backbone^23^,^37^,^38^,^39^, and further result in DNA compaction via protein–protein interactions^9^,^14^,^40^,^41^. However, in the present study, we found that circular plasmid DNA mechanically stressed by local torque rearranged its conformation into a balanced dual-toroidal-spool conformation (Fig. 1). The accumulation of ~11.7 to 37.6 local torques per 4,361 bps plasmid DNA introduced a global transition of DNA conformation from a random supercoiled structure to an eight-shaped dual-spool configuration over a time scale of 3–5 hours. Theoretically, canonical B-form DNA retained the lowest energetic state, in which minimal mechanical stress exists. When DNA was bent or twisted, it responded as a stiff, springy molecule in conformational flux until a new low energetic state was achieved. The auto-packaging behaviour of plasmid DNA in response to torque mechanical stress from small molecules, such as the platinum complex, was reported and investigated for the first time in the present study. These findings could provide new clues for understanding the mystery of DNA auto-compaction in bacterial cells.

Bacterial chromosomal DNA is highly compacted into a nucleoid. Nucleoid DNA is considered a highly mechanically stressed object whose intrinsic mechanical features play governing roles^17^,^42^. The findings of our PALM studies strongly support this opinion (Figs 3 and 4). Time sequential evolutions of HNS clusters both in slow and fast growing *E. coli* cells demonstrate their unique regular patterns during cell cycles, indicating the existence of unknown factors which govern these evolutions. In particular, characteristic HNS cluster pairs at a relative constant distance (~0.88 μm) were readily observed in new born cells and early growing cells (0–30 min) under slow growing conditions. Over this time span, the cells are known to contain approximately one copy of chromosomal DNA. Subsequently, these HNS pairs developed into three clusters and then became two new clusters again during the late stage of DNA replication and after DNA replication (60–100 min). The new HNS pairs have a significantly different distance value (i.e., 1.41 to 1.65 μm). The cells over this time span are known to contain approximately two copies of chromosomal DNA. The specific “two-three-two-four” mode of HNS cluster evolution during a cell cycle under slow growing conditions, and the “one-three-two” mode of HNS cluster development within a cell division cycle in cells under fast growing conditions, are observed regularly in growing *E. coli* cells. These experimental data agree well with our model of chromosome organization in *E. coli*. Based on AFM single molecular observations and PALM evaluation, we have explained a novel organization pattern for circular chromosomal DNA in living *E. coli* cells (Fig. 2). However, due to the limitations of experimental techniques, we cannot prove our model by direct observation of circular DNA compaction by NAPs *in vivo* and/or *in vitro*. We leave this problem open for future investigations to discover more about the mechanisms underlying chromosomal DNA compaction. From the findings in the current study and previous reports, we propose that NAP binding introduces mechanical stress to chromosomal DNA such that stressed chromosomal DNA rearranges its conformation into one docked spool conformation (Fig. 4) or a dual-toroidal-spool conformation (Fig. 3, i and vi) depending on the isolate state of DNA. Our findings indicate that the unique intrinsic physical properties of DNA might be the determining factor underlying auto-packaging behaviours of these giant biomolecules in cells.

We tested the ability of our model to accommodate previously published experimental data. It has been difficult to directly observe the compaction pattern of DNA in a nucleoid due to molecular crowding in live bacteria and the loss of the original morphology of the nucleoid after cell lysis^17^,^43^. However, in recent years, a number of well-designed investigations have provided insight into the dynamic organization of the nucleoid in living *E. coli*^2^. Surprisingly, a majority of these experimental results support our model for dynamic chromosomal organization in *E. coli* during a division cycle. For example, six macrodomains representing a higher order of nucleoid organization coincided with the temporal and spatial distribution of DNA strands at significantly different fluctuating states, as described by our model (Supplementary Discussion)^15^,^44^. The distribution pattern of pairs of chromosomal loci was easily explained by the dual-toroidal-spool configuration^45^,^46^. Our model accommodates the possibility that one copy of chromosomal DNA bears two HNS clusters exactly located at cell quarters^7^, as well as the asymmetry of the newly split sister nucleoid^42^,^47^. The 3D model of the rotational dual-toroidal-spool with an upside down relationship between the two spools explains the helicity of the nucleoid without apparent handedness (Supplementary Discussion and Supplementary Fig. S7)^17^,^35^. Our findings prove that sister nucleoids are basically non-intermingling objects that are separated from each other in a single coordinated transition with global chromosomal movements^17^,^42^,^47^. Overall, our observations provide the direct experimental proof of the auto-packaging behavior of circular plasmid DNA upon unwinding and bending mechanical stress by the platinum complex. The proposed model for dynamic compaction of the nucleoid may expand our understanding of the mechanisms and pathway of chromosome organization in *E. coli*. The present study provides new perspectives for investigating fundamental mechanisms of chromosome organization and gene regulation in bacteria and eukaryotes in the future.

Finally, let us forget the impression that DNA is a loosely relaxed string with local bends by proteins.

Let us appreciate DNA as an elastic, rigid (in nanometer-scale), fluctuant biomolecule which tends to auto-package onto itself with the aid of chaperone NAPs.

Though no special physical or chemical laws in cells, we cannot help wondering, adoring and admiring how well organized a cell can be.

### Methods summary

Atomic force microscopy analysis was performed essentially the same as described previously^20^. Bacterial strain construction of the chromosomal fusion HNS-mEos2 MG1655 strain was performed according to previous methods^7^,^48^. Detailed procedures for imaging and data processing are available in the Supplementary Methods.

## Additional Information

**How to cite this article**: Liu, Y. *et al.* A model for chromosome organization during the cell cycle in live *E. coli.* *Sci. Rep.* **5**, 17133; doi: 10.1038/srep17133 (2015).

## Supplementary Material

Supplementary Information
