# Supplementary material for: A model for chromosome organization during the cell cycle in live E. coli
Source: Sci Rep. 2015 Nov 24;5:17133. doi: 10.1038/srep17133 (PMC4657085; doi:10.1038/srep17133)
Supplement: Supplementary Information [file srep17133-s1.pdf]

Supplementary Information

A model for chromosome organization during the cell cycle in live *E. coli*

Yuru Liu*, Ping Xie, Pengye Wang*, Ming Li, Hui Li, Wei Li, Shuoxing Dou

Key Laboratory of Soft Matter Physics, Beijing National Laboratory for Condensed Matter Physics, Institute of Physics, Chinese Academy of Sciences, Beijing 100190, China

Supplementary Figures

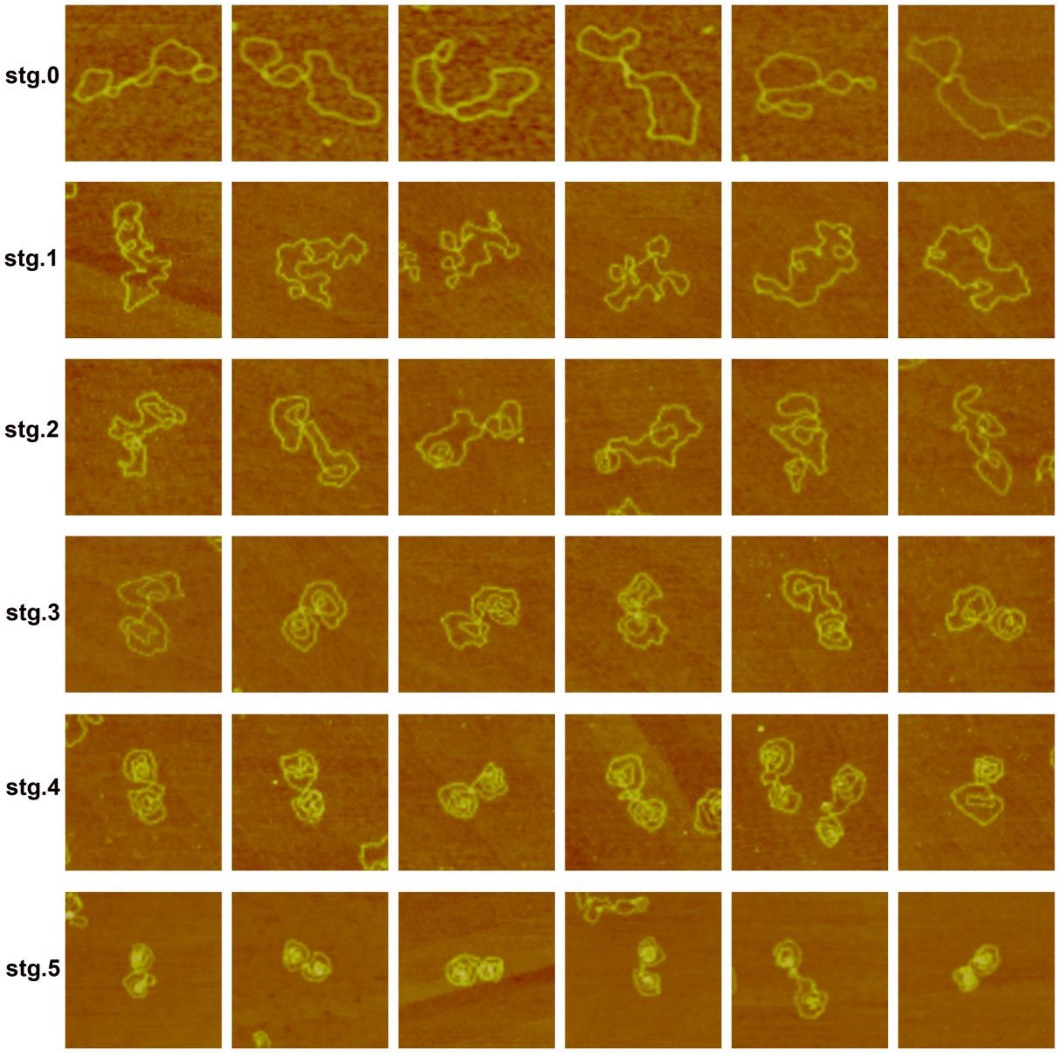

Supplementary Fig. S1. Montage of AFM images of DNA molecules in different stages of the formation of dual-toroidal-spool conformation. The development of intact supercoiled plasmid DNA to dual-toroidal-spool conformation involves five stages in bulk. **stg.0**, Intact plasmid DNA; **stg.1**, local small toroidal supercoils formed in DNA; **stg.2**, local irregular toroidal spools with different sizes; **stg.3**, loose two toroidal spools with roughly comparable engulfment of DNA contents; **stg.4**, compacted dual-toroidal-spool with symmetric conformation; **stg.5**, tightly wrapped dual-toroidal-spool with a condensed centre core. The images are 0.5 μm in width.

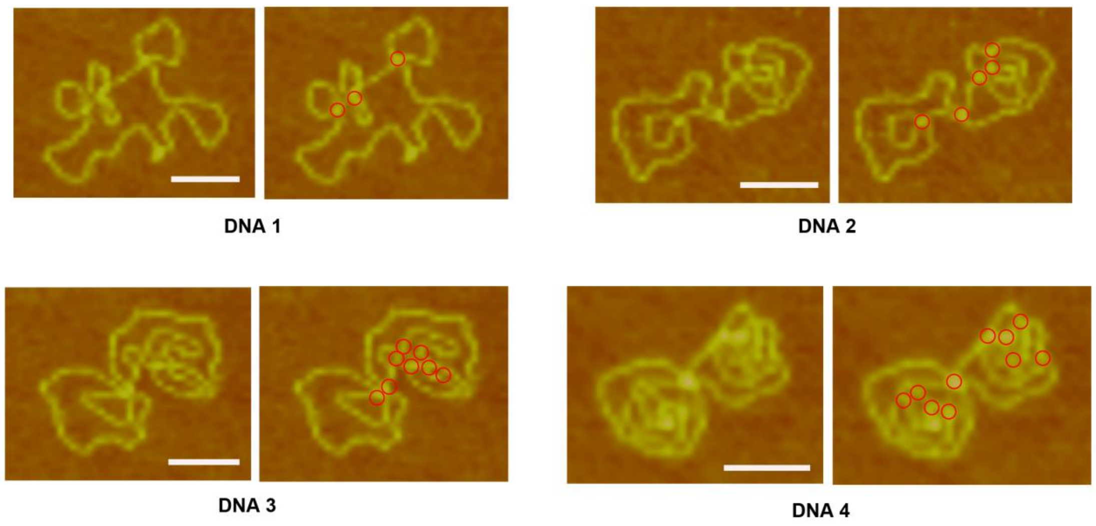

Supplementary Fig. S2. Identification of writhe information on DNA molecules. The writhe is defined as crossing of two DNA fragments in our study. The AFM images of DNA molecules (i.e., DNA 1 to 4, at different stages of DNA compaction) were enlarged and the DNA backbone was tracked. The writhe points where two DNA fragments cross each other were recorded by red circles. Scale bars are 100 nm.

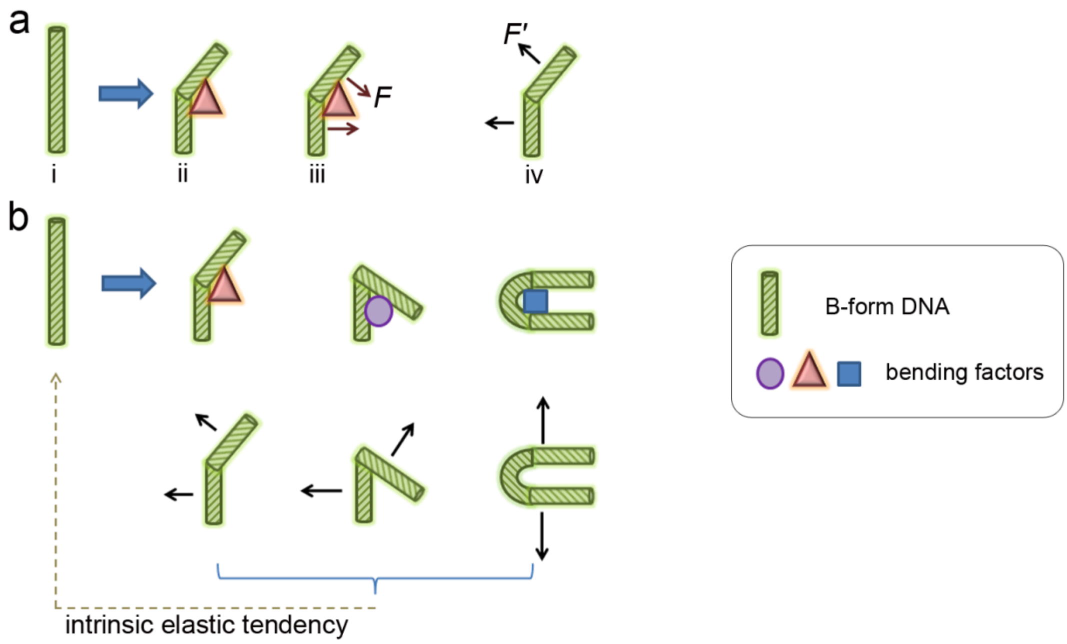

Supplementary Fig. S3. Diagram of DNA response to bending mechanical stress. **a**, The canonical B-form DNA has the lowest energetically mechanical state in which minimal mechanical stress exists (i). When a normal B-form DNA was bent by external bending factors, bending factors generated action *F* (brown arrow, iii), which was overall perpendicular to DNA strands and pointed to the side of bending factors; correspondingly, deformed DNA generated reactions *F* ´(black arrow, iv) with an opposite direction to *F*. **b**, *F* and *F* ´increased with increasing DNA bending. The bent DNA demonstrated a tendency to return back to B-form DNA because of the intrinsic elastic property of helical DNA.

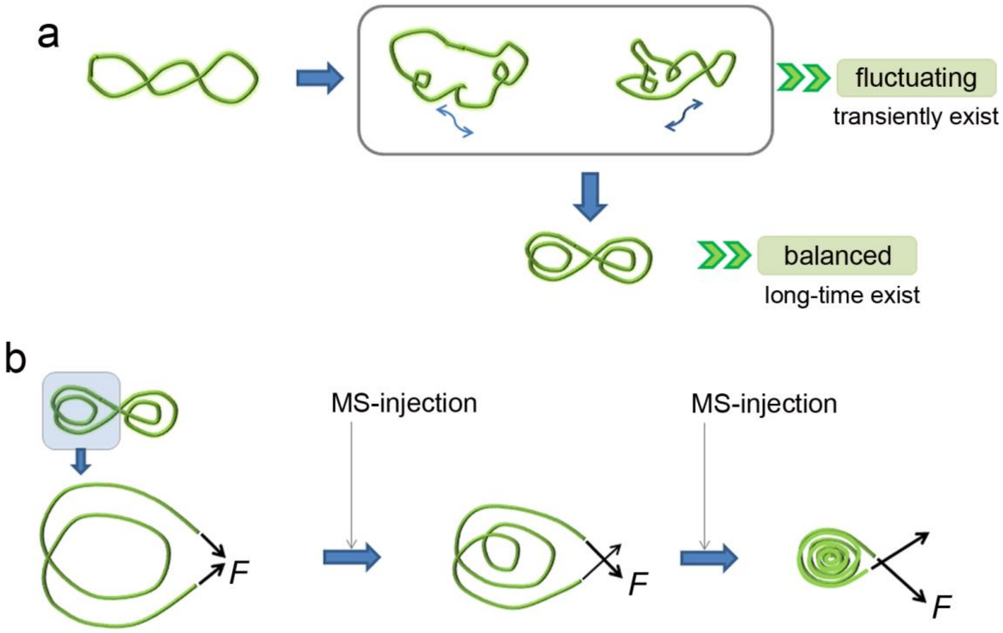

Supplementary Fig. S4. Diagram of the mechanism for circular DNA to compensate sharp bending and untwisting mechanical stress.

a, Diagram to explain the mechanism for the formation of DNA spools in mechanically stressed circular DNA. When DNA was bent or untwisted by binding factors, local strands were deformed. Thus, the deformed part was mechanically stressed to obtain a higher energetic state. The uneven distributed tension and mechanical stress along the DNA backbone resulted in fluctuation. Fluctuation continued to propagate along the DNA backbone, and additional twisting mechanical stress could not be released from the ends of DNA as linear DNA does. When nearly spherical or ellipsoid curvature of DNA strands appeared, deformation was most evenly distributed along DNA, so fluctuation decreased and DNA was likely to be frozen into this conformation. Therefore, mechanically stressed DNA fell into a new lowest energetic state in which fluctuation along the DNA chain was mostly reduced by the self-balancing effect and evenly distributed curvature of DNA. For a circular DNA, a DNA spool was a more stable structure than local toroidal supercoils (inside the grey solid line box).

b, Diagram illustrating the coiling process of DNA spool in mechanically stressed circular DNA. The increasing untwisting effect by external factors on circular DNA continued to disturb DNA. DNA compensated for these effects by increasing its writhe number. Thus, DNA became more tightly compact to accommodate the increasing writhes, i.e., new DNA circles. MS injection means mechanical stress injection. *F* is the tension along DNA, which increased with the accumulation of torque injections. *F* serves as the supporting force to act on the remaining spool.

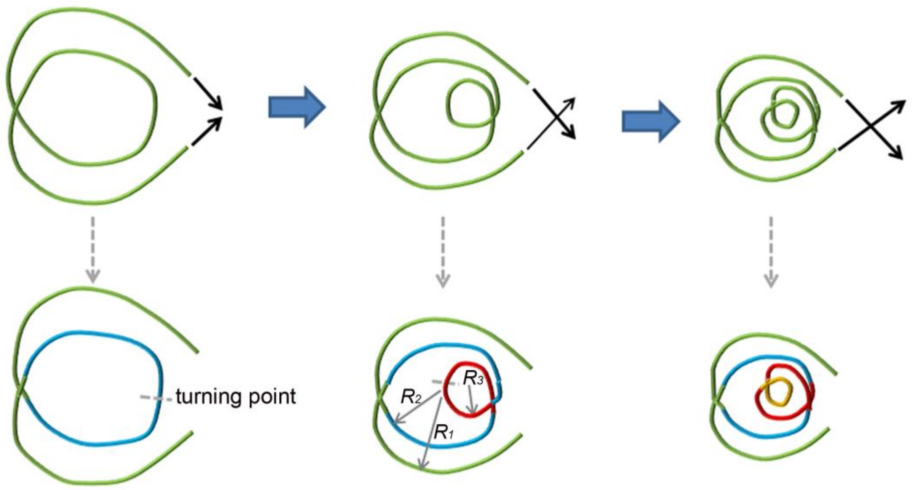

Supplementary Fig. S5. Diagram of DNA coiling onto itself to accommodate new writhes upon mechanical stress injection. New writhes accompanied with new DNA circles were introduced into the DNA spool to compensate for untwisting mechanical stress. In the lower panel, the new circles of DNA are drawn in different colours for clarity. One half of DNA circle is entering DNA strand and the other half is exiting DNA strand. The results of AFM analysis show that the circles became significantly smaller from the periphery to the centre of the spool. In this case, the bending energy of each circle increased from the periphery to the centre core of the spool.

To estimate the bending energy of each DNA circle, linear elastic theory of a linear spring was applied (as described in a teaching program in Massachusetts Institute of Technology, http://www.mit.edu/~kardar/teaching/projects/dna_packing_website/bending.html).

The bending energy is of the form E = 1/2 $k\,x^2$,

The energy to bend the DNA chain is of the following form,

$$\mathrm{E} = \frac{1}{2}kL\left(\frac{dt}{ds}\right)^2$$

where dt/ds is the derivative of the tangent vector to each simulated DNA Kuhn segment of the DNA chain. We simplified each DNA circle being bent into a circle with radius R, dt/ds = 1/R. *L* is the length of DNA. Thus, the bending energy of each DNA circle is expressed as follows,

$$\mathrm{E} = \frac{1}{2}kL/R^2 = \frac{1}{2}k(2\pi R)/R^2 = {}^{k\pi}/_{R}$$

where k is $\xi_P k_B T$ and ξ_p is the persistent length of DNA. The bending energy increases with the shortening of radius of DNA circles.

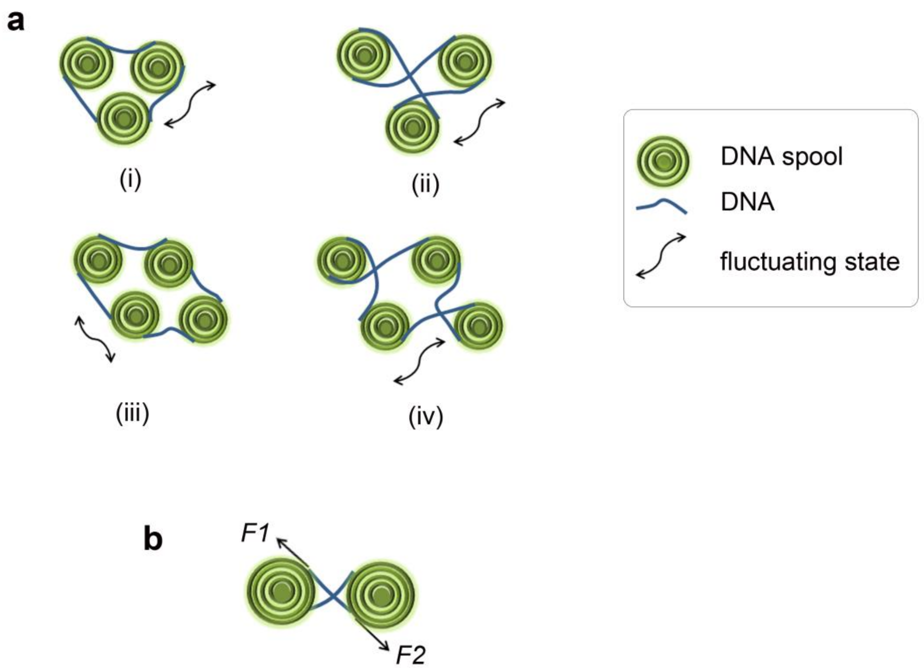

Supplementary Fig. S6. Diagram of the mechanism for dual symmetry predomination in mechanically stressed circular DNA molecules. **a**, If three or more comparable local DNA spools (green circles) formed spontaneously inside a circular DNA, whether faced to one side (i, iii) or both sides (ii, iv), the odds for occurrence of a moment when the tension along all the hinge DNA strands (blue lines) reaches an exactly balanced state would be very rare. Thus, significant fluctuation continuously propagated inside the circular DNA molecules until a balanced structure formed.

b, In a fluctuating DNA molecule, the tension *F1* generated from one developing spool acting on the hinge part dynamically changed its direction and value, similar to *F2*. In a simplified estimation, if we assume that the possible distribution pattern for *F1* and *F2* pair is N, then the balanced state is unique (where *F1* = *F2* with exact opposite directions between *F1* and *F2*). The odds of occurrence for one DNA section at the hinge part in a balanced state is: $C_N^1 = 1/N = N^{-1}$.

Thus, the odds for a balanced dual-spool occurrence is as follows: $R_d = C_N^1 \times C_N^1 = N^{-2}$, where the two crossing DNA sections are required to reach a balanced state simultaneously.

The odds for a balanced triplex-spool occurrence is as follows: $R_t = C_N^1 \times C_N^1 \times C_N^1 = N^{-3}$;

the odds for a balanced quadruplex-spool occurrence is: $R_q = C_N^1 \times C_N^1 \times C_N^1 \times C_N^1 = N^{-4}$.

In fact, R_t and R_q may even be lower than the estimation above because the spontaneous occurrence of comparable triplex-spool or quadruplex-spool is rarer than dual-spool in solution.

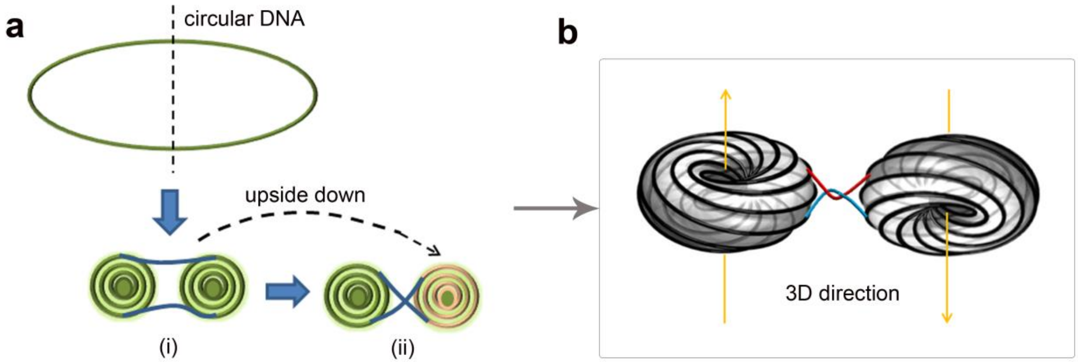

Supplementary Fig. S7. Diagram of 3D spatial relationship between two spools in dual-toroidal-spool conformation. **a**, When circular DNA was bent and untwisted by NAPs, the locally developed spools shared similar structural features (i). This phenomenon was due to the same mechanical effects introduced by the same types of NAPs into different parts of circular DNA. Each spool (green, i) bore similar handedness, and both were negative supercoiled toroidal spools because of the untwisting effects of NAPs. In reality, conformation (i) does not exist. Given the intrinsic rotational tendency of negative supercoils of DNA, conformation (i) rotates into conformation (ii) with a crossover hinge part. **b**, Therefore, the two spools inside a dual-toroidal-spool retained a substantially upside down spatial 3D relationship between them. The image of the DNA spool (black) was copied and adapted from reference [1].

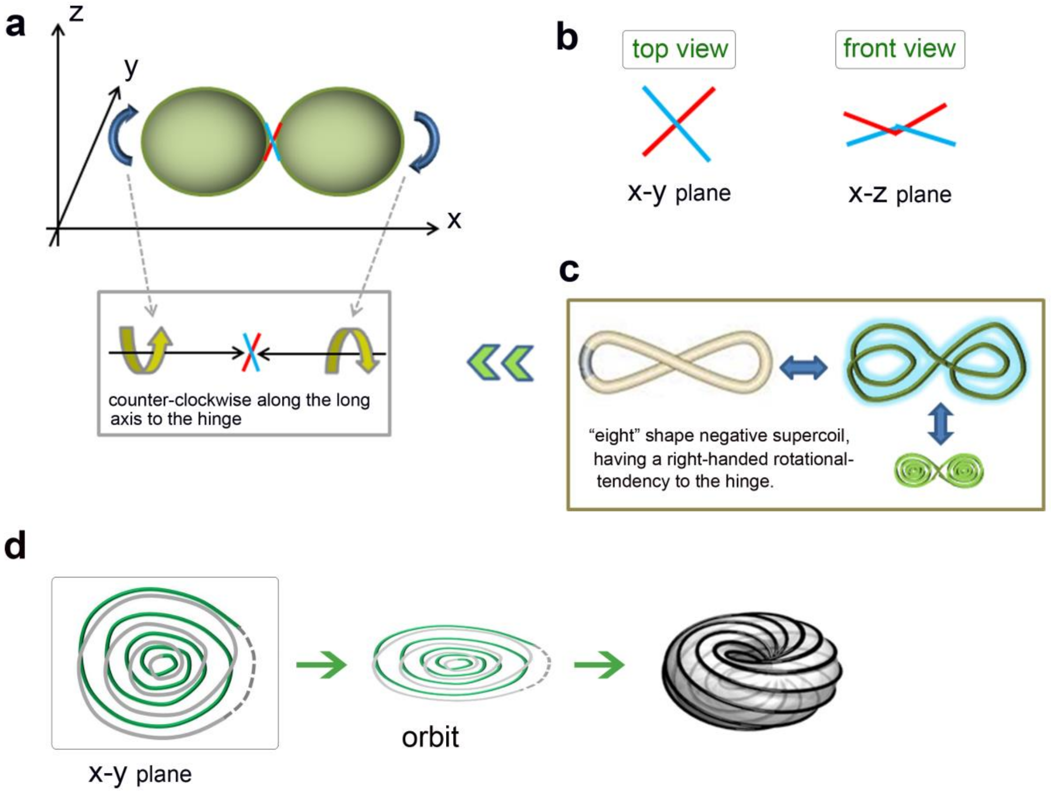

Supplementary Fig. S8. Diagram for structural details of dual-toroidal-spool conformation. **a**, Two DNA ellipsoids connected with a hinge was basically the 3D morphology for the dual-toroidal-spool conformation. Each spool had the same 3D rotational tendency along the long axis to the hinge (blue arrows), and shared the same structural features. **b**, Structural features of the hinge of the dual-toroidal-spool conformation. They crossed over with each other but were not intertwined. Each spool rotated on the hinge in the middle between them, buckling the two DNA strands (blue and red) of the hinge together. Left panel, the projection of the hinge onto the x–y plane. Right panel, the projection of the hinge onto the x–z plane. **c**, The dual-toroidal-spool is essentially an eight-shaped negative supercoil. In negative supercoils of circular DNA, DNA strands retain right-handed rotational tendency. Therefore, each spool in (**a**) has a counterclockwise rotational tendency along the long axis to the hinge. The DNA image in the left panel was adapted from reference [2]. **d**, 3D structural feature of the DNA spool. DNA strands in each spool are wrapped with almost evenly distributed curvature, with the projection of DNA strands onto the x–y plane (left) serving as an orbit (middle). In 3D solution, the DNA strands in spool rotated along the orbit to compensate for the bending stress to form an ellipsoid rotational spool (right), as elucidated by a previous theoretical investigation [1]. The dashed line indicates linking to the hinge (left and middle).

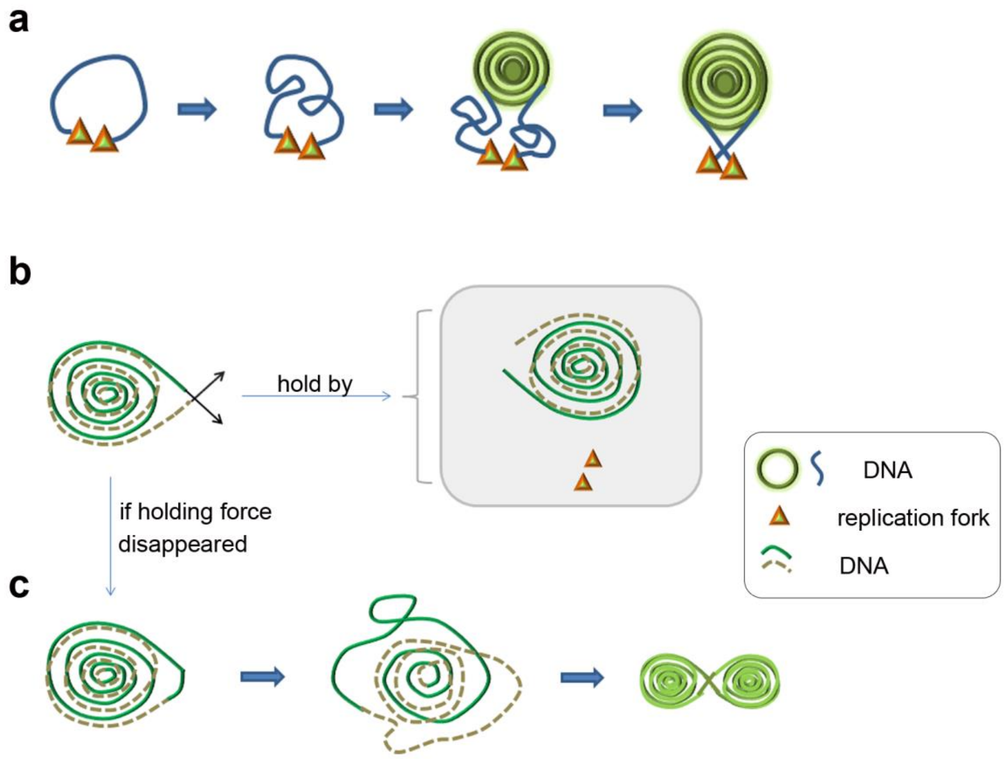

Supplementary Fig. S9. Diagram of mechanism for docked spool formation during cell division cycle. **a**, Newly synthesised DNA (blue) docked on two replication forks was bound by NAPs, which are abundant in the cytoplasm of *E. coli*. The bindings of NAPs and other replication-associated proteins introduced mechanical stress and torsional constraints into replicating sister DNA, so the mechanically stressed sister DNA auto-wrapped into a single-spool conformation held by replication factories and achieved the lowest energetic mechanical state (green).

b and **c**, The underlying mechanism is that the single-spool is in a lower energetic state than the dual-spool when both of them developed from the same DNA molecule. Considering that the overall radius of DNA circles distributed in a single-spool is bigger than that in a dual-spool, the total bending energy of DNA single-spool is lower than that of DNA dual-spool. Therefore, a single-spool is more energetically favourable than multiple-spools for DNA to settle down. In a single-spool, the holding force either from replication factories or other comparable spools provided an external force to hold itself in shape. Without a holding force, the isolated single-spool spread outwards because of the intrinsic elastic property of helical DNA; thus, the spool dissembled. Finally, a dual-spool formed because of the self-balancing effect for governing DNA to fluctuate into the lowest energetic state. Although a dual-spool has a higher energetic state than a single-spool, it is the lowest energetic structure among other possible conformations of mechanically stressed circular DNA (except single-spool). Dual-spool demonstrates good self-balance, so it is most favourable for an isolated circular DNA to be frozen into among other possibilities as mentioned in Supplementary Fig. S6.

Supplementary discussion

The organization of nucleoids in live *E. coli* remains largely unknown even though *E. coli* has been used as a classical model organism under extensive investigations by various molecular genetic approaches for the past decades. With the application of new techniques available in recent years, our understanding of the features of nucleoids has greatly improved [3-5]. Although chromosome DNA in bacteria is highly compacted to fit into a confined cytoplasmic volume, nucleoids possess a soft nature that differs from viral DNA tightly compacted in a capsid [6]. Nucleoids do not always fill out all the cytoplasmic space, and they move as a solid without changing much of their shape and volume inside the cytoplasm [7, 8]. The nucleoid can even retain part of their compactness over a time scale of tens of minutes after they are isolated from lysed cells [6, 9]. These observations suggest a soft but solid nucleoid, which is rather in a highly organized form than in random coils.

E. coli lacks a mitotic system and an equivalent prokaryotic mechanism to separate sister chromosomes. There is no defined special cytoskeletal apparatus or docking proteins on the inner membrane to mediate the segregation of sister DNA [10, 11]. Bates et al. have shown that loss of the adhesion of sister nucleoid triggers global chromosome movements and mediates chromosome segregation, demonstrating a unique feature for bacterial sister chromosome segregation [12]. In the present study, we proposed a model to elucidate organization and segregation of chromosome DNA in live *E. coli* during a cell cycle. The model accommodates the thus-far-discovered remarkable features of nucleoids in vivo. We focus the discussion in the following six sections.

1. Six MDs of nucleoids display their particular spatial and temporal distribution of DNA contents with varied mobility during cell cycles.

Higher order organization of bacterial chromosome is poorly understood. The organization of the chromosome into MDs was first discovered by Niki and colleagues based on FISH observations [13]. The MDs Ori and Ter were defined as the 1 Mb regions flanking the *oriC* or *dif* chromosomal locus. All the chromosomal loci in Ori MD or Ter MD have their respective unique localisations, which are distinct from the rest of the chromosome. Four MDs and two less-structured regions were further observed by analyzing the recombination efficiency using a site-specific recombination system [14]. Two of the MDs defined by recombination efficiency, which were similar to the Ter and Ori MDs, were Right and Left MDs, respectively. Two less-structured regions flanking the Ori MD were named NSR and NSL MDs (Supplementary Discussion Diagram 1a). The recombination efficiency in NSR and NSL MDs was much lower than that in the other four MDs.

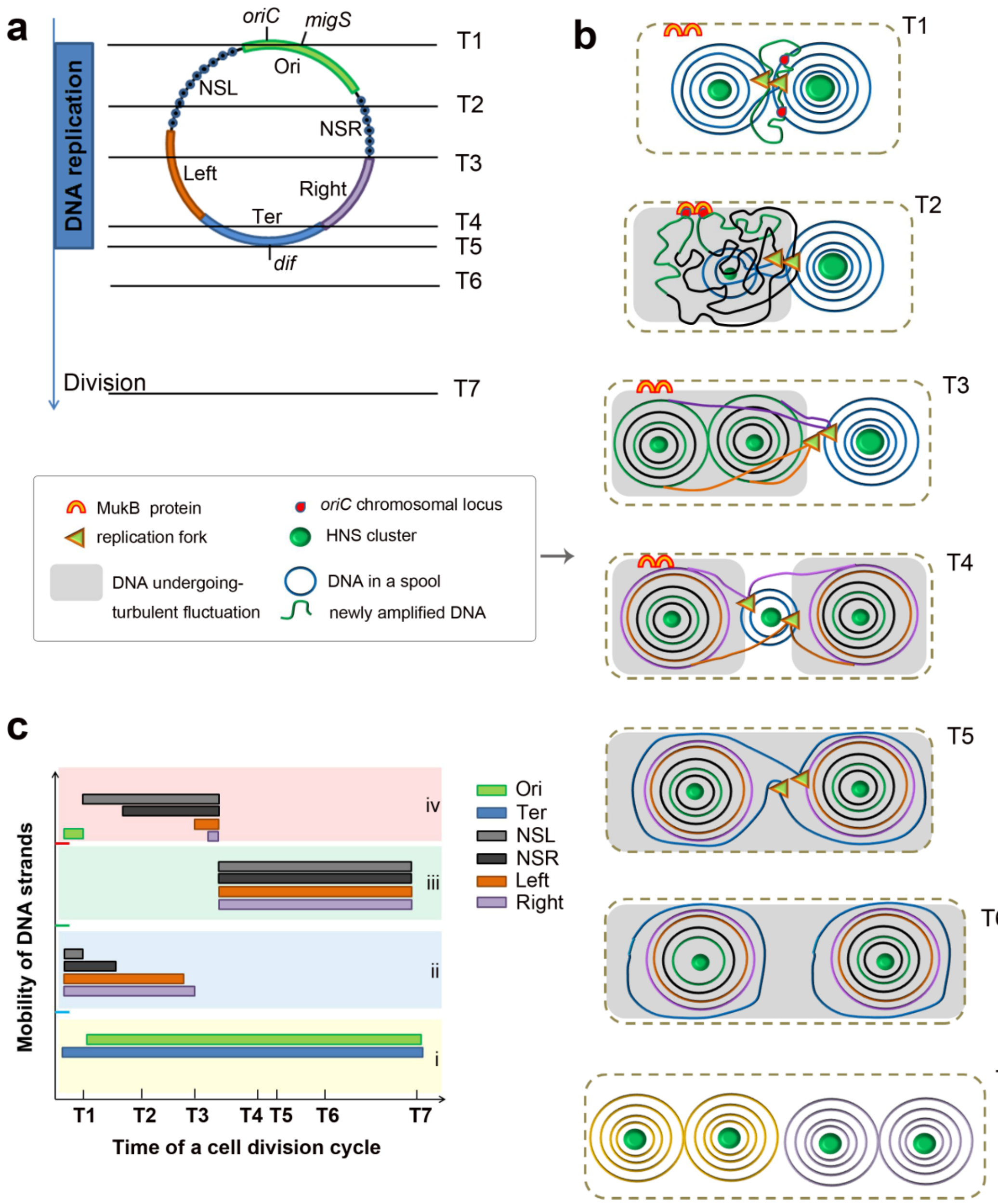

Supplementary Discussion Diagram 1. Mobility features of DNA strands in six MDs elucidated by our model. (a) Map of MD distribution on *E. coli* chromosome according to a previous description [14] and the time points selected in a cell cycle. The green region containing *oriC* and *migS* is Ori MD. The black regions flanking Ori MD are NSL and NSR MDs. The blue region is Ter MD, and it is flanked by Right (purple) and Left MDs (orange). **(b)** In our model, the *migS* locus was amplified at T1 and then docked onto cell quarters. The ~1 MB DNA flanking this locus was also docked onto cell quarters [15, 16]. The NS MDs underwent free diffusion for a significant time span, and they were randomly disposed in the cytoplasm (from their replication to ~T3). At ~T3, thus-far-replicated sister DNA collapsed into a DNA spool docked on replication factories. From ~T3–T6, newly amplified DNA strands briefly underwent free diffusion, and wrapped on the developing spools where fluctuation was significant. At T7, the maturation of dual-toroidal-spool reduced fluctuation by achieving a self-balanced and low energetic mechanical state. **(c)** The fluctuation state of DNA from each MD during a cell cycle was scored into four levels. Level

i, constraint by cytoplasmic components; level ii, fluctuated in a mature DNA spool; level iii, fluctuated in a developing DNA spool; and level iv, free diffusion.

The cytological and molecular mechanisms underlying MD development are currently unknown. Based on the observations in the present study, the spatial and temporal distribution and organization of chromosome DNA during a cell cycle were proposed (Supplementary Discussion Diagram 1b). In brief, each DNA spool occupied half of an *E. coli* cell. When replication of chromosome DNA was initiated, the *oriC* locus was first pulled to pass through the replication factory located at the centre of the cell. Newly synthesised *oriC* and the loci in Ori MD were then tethered and disposed at cell quarters by MukB protein and *migS* cis element, as revealed previously [15, 16]. Thus, Ori MD was diffusion constraint. The replicated DNA region outside Ori MD in the spool underwent free diffusion for a significant time until the formation of two additional new spools at around the middle stage of DNA replication. These DNA regions corresponded to the NSL and NSR MDs flanking Ori MD. The DNA contents located in the Right and Left MDs were replicated. The newly amplified DNA fragments from the Right and Left MDs underwent a brief period of free diffusion, and wrapped onto the already formed docked spools. Thus, free diffusion was minimal for Ori, Right, Left, and Ter MDs, but significant for NS MDs.

During a cell division cycle, DNA fragments in nucleoids are mostly in a dynamic fluctuation state at different levels for a special time span (Supplementary Discussion Diagram 1c). We scored the fluctuation state based on four levels. The lowest level (i) is diffusion constraint by the docking system to associate with other cytoplasmic components. The second level (ii) involves DNA fluctuation on mature dual-toroidal-spool without local docking systems. The dual-toroidal-spool is a well-balanced conformation in the lowest energetic state, which allows DNA to freeze to stop significant fluctuation. In the third level (iii), DNA fluctuates on a newly formed, dynamically fluctuating, non-balanced spool. The highest level (iv) is free diffusion. DNA in this level is not packaged yet. In particular, NS MDs spend almost the entire cell cycle to achieve a higher fluctuation state (iii and iv). They are the only MDs that have to freely diffuse for a significant time during a cell cycle. This feature might explain why they are regarded as less-structured regions in previous observations. By contrast, Ori and Ter MDs are in the lowest fluctuation state (i) for almost the entire cell cycle by associating with MukB or MatP, respectively, and they are docked on defined cytoplasmic positions [16, 17]. Right and Left MDs are in a relative median state of fluctuation (ii and iii) in which DNA is organized into mature or developing spool. Our model was in agreement with the results of a previous study presenting the diffusion speeds and travel distance of chromosomal loci in each MD in *E. coli* [18] (Supplementary Discussion Published Data 1). The highest mobility and greatest travel distance were observed for the loci in NS MDs in their study. The DNA fluctuation state coincident with homologous recombination efficiency would be an interesting issue to be further addressed for deeper understanding of mechanism for recombination.

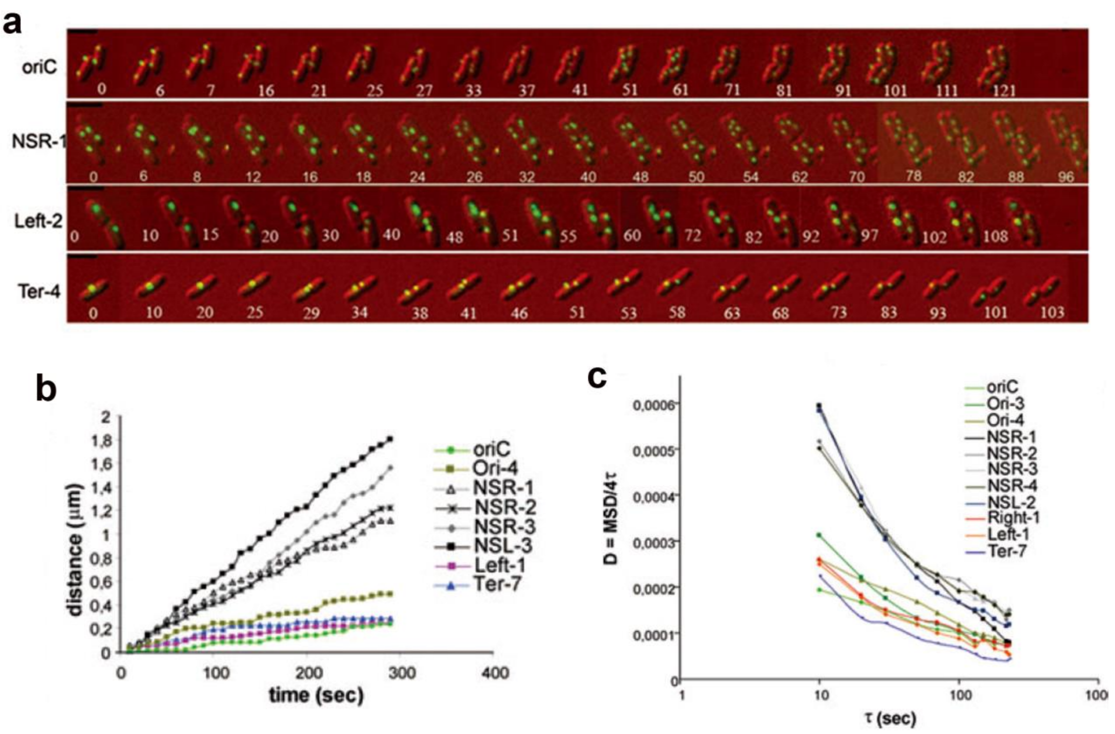

Supplementary Discussion Published Data 1. Mobility features of DNA strands in six MDs [18]. (a) Fluorescent chromosomal loci distribution during a cell cycle. NSR-1 exhibited the longest travel distance in the cytoplasm among the four chromosomal loci. **(b)** The travel distance of chromosomal loci in different MDs. **(c)** The diffusion speeds of chromosomal loci in different MDs.

The distribution of MDs on the chromosome map presents several geometric features. The detailed distribution lengths and positions of MDs on the chromosome map indicate the subtle relationship between MDs and DNA replication (Supplementary Discussion Diagram 2). Firstly, two NS MDs had different sizes and Ori MD was not centred on the *oriC* locus. Meanwhile, the lengths of DNA fragments from *oriC* to the ends of each NS MDs were largely comparable (O-N1 and O-N2 fragments, Supplementary Discussion Diagram 2). This geometric feature might reflect the sudden collapse of newly synthesised sister DNA into more stable conformations, thereby preventing the free diffusion of DNA contents replicated after NS MDs to end a ‘non-structured’ state of those DNA parts. Considering the replication initiating from *oriC* and preceding bidirectional with the same speed, the comparable lengths of O-N1 and O-N2 indicate a subtle relationship with DNA replication. Secondly, the length of NSR + Ori + NSL was barely equal to that of Right + Ter + Left on the chromosome map, which coincides with the time of collapse of newly synthesised DNA into docked spools at the middle stage of DNA replication. In this paper, we propose new perspectives regarding DNA fluctuation and replication to explain the origin of MDs of nucleoids, which might provide new clues for further investigations.

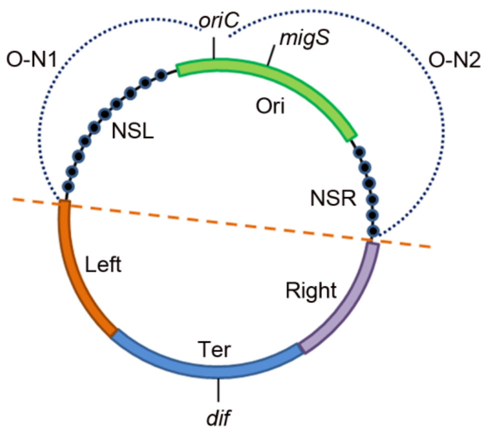

Supplementary Discussion Diagram 2. Geometric features of six MDs in the genetic map of *E. coli*. **(a)** Geometric features of MD distribution in the chromosome map subtly indicate the relationship between MDs and DNA replication. Firstly, the length of *oriC* to each end of NS MDs (i.e., O-N1 and O-N2 fragments) was roughly comparable. Secondly, NSL + Ori + NSR fragment was barely equal to Right + Ter + Left fragment.

2. Left and right chromosome arms are organized in separate cell halves stochastically.

The right and left chromosome arms of *E. coli* are located in different cell halves, as observed in fluorescent loci pairs on separate arms of the chromosome lying in separate halves of a cell [19, 20]. Our model explained this phenomenon as the loci pairs distributed in different spools in a nucleoid. Furthermore, our model also accommodated the contradictory data in each group that ~10%–20% cells exhibited reversed distribution patterns to the major pattern in previous studies (Supplementary Discussion Published Data 2c). We attempt to explain the underlying mechanism as follows. After completion of DNA replication, one docked spool containing one sister chromosome DNA re-arranged into dual-toroidal-spool conformation. In this transition, nearly half of the DNA contents of chromosome coiled into each spool of the dual-toroidal-spool conformation. Re-arrangement occurs under conditions when Ori MD and Ter MD are associated with cellular components, because Ori MD is located near cell quarters and Ter MD is in the centre of the cell [13, 15, 16, 21]. Thus, the two domains were likely distributed in separated spools because of spatial constraints (Supplementary Discussion Diagram 3). Other parts of chromosome DNA isolated in the cytoplasm could be coiled into either spool. Theoretically, numerous possibilities exist for a circular chromosome DNA to be engulfed into nearly equal halves (along dashed lines, Supplementary Discussion Diagram 3). In reality, given the initial coiling of newly replicated Ori + NSL + NSR into a docked spool, followed by gradual wrapping of Right + Left + Ter MDs, the confinement of cellular space, crowding of cytoplasmic molecules, association with cytoplasmic components and influence of the stochastically binding of NAPs, etc., there might be preferred

pathways for binary engulfment of DNA contents into dual-spool in dividing *E. coli* cells. The pathway is predicted to be energetically favourable and biochemical stochastically. In the present study, we could not address and investigate such an issue. Based on the published experimental data [19, 20] (Supplementary Discussion Published Data 2), there seems to be a bias of binary engulfment of chromosome DNA contents along the axis largely across Ori and Ter directions rather than along the axis across NS MDs or Right and Left MDs directions. This would be an interesting issue to be investigated in future studies to understand more about the pathways of chromosome packaging in live *E. coli*.

Balanced conformation is preferred for a final stable 'frozen' nucleoid, so nearly half of DNA was engulfed into one spool of the dual-toroidal-spool conformation. The loci pairs near each other in the chromosome map were at higher odds to be distributed in the same spool than in a separate spool as checked from a cell population, which was consistent with the experimental observations (Supplementary Discussion Published Data 2a–c). However, close pairs may be engulfed into different spools, so small portions of cells retained different patterns of loci dispositions to the major portions of cells (Supplementary Discussion Published Data 2c). Interestingly, the loci pairs which were separated about 180 ° on the circular chromosome, though farther away in terms of left-arm-to-right-arm distance than other pairs separated ~120 ° on the genetic map, displayed higher occurrence rate to be engulfed into same spool than that for 120 ° pairs, probably due to 180 ° loci pairs lying near edges of one half chromosome DNA [19].

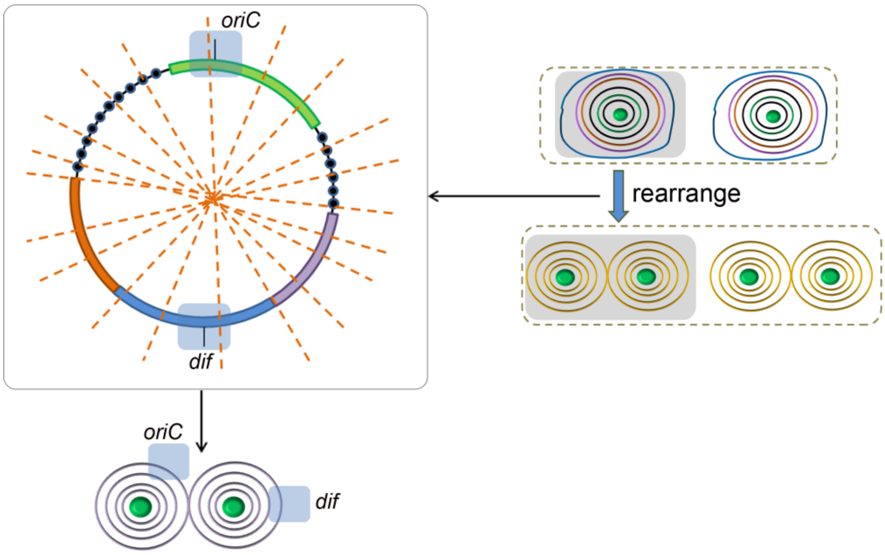

Supplementary Discussion Diagram 3. Pathways for stochastic rearrangement of single-spool containing one sister chromosome DNA into a dual-toroidal-spool conformation. Two immobilised chromosomal regions (Ori and Ter MDs) exist in *E. coli*. When a single-spool rearranged into dual-spool with comparable size, the binary engulfment pathways (orange dashed lines) occurred stochastically under the influence of cytoplasmic events.

a

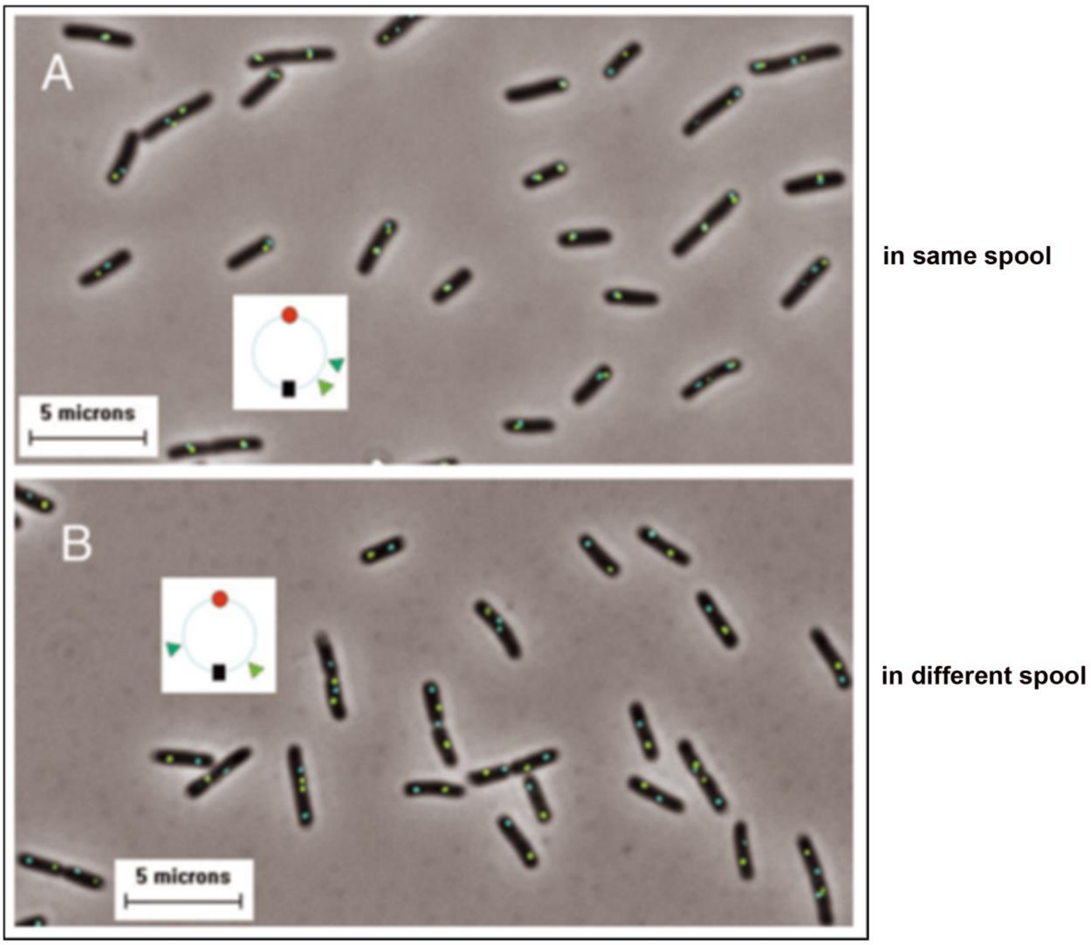
A
5 microns
B
5 microns
in same spool
in different spool

b

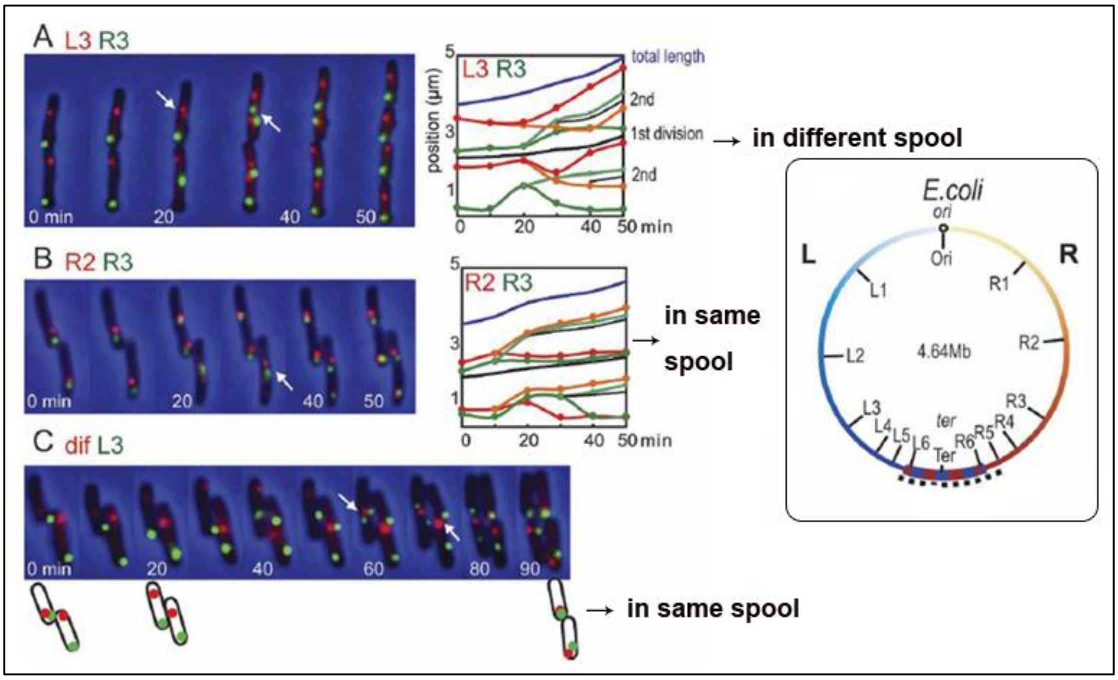
A L3 R3
0 min
20
40
50
L3 R3
total length
2nd
1st division
2nd
position (μm)
50 min
→ in different spool
B R2 R3
R2 R3
→ in same spool
C dif L3
0 min
20
40
60
80
90
→ in same spool
E.coli
ori
Ori
L
R
L1
R1
L2
R2
4.64Mb
L3
L4
L5
L6
ter
Ter
R6
R5
R4
R3

c

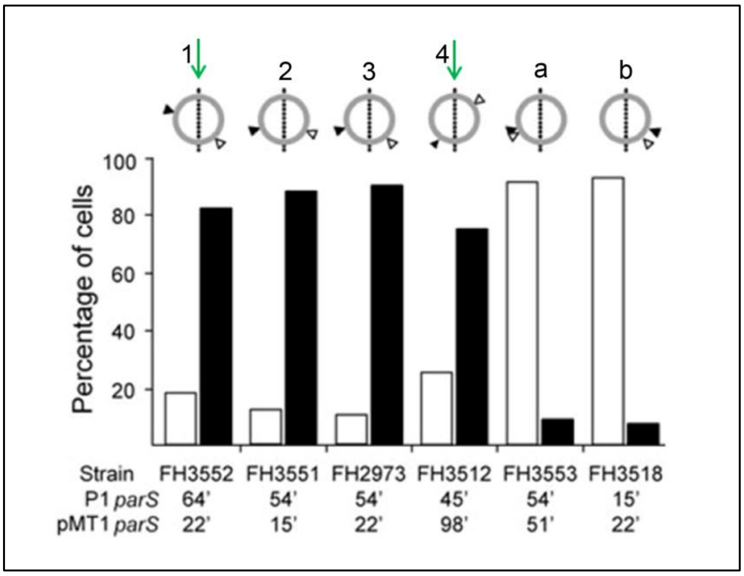

Supplementary Discussion Published Data 2. Distribution pattern of chromosomal loci pairs in cell halves [19, 20]. (a, b) Occurrence of chromosomal loci pairs located in the same spool or different spools, which was correlated with their distance in the chromosome map. The loci pairs near each other exhibited high occurrence in the same spool, whereas the pairs far from each other displayed high occurrence in different spools. **(c)** In each group, the majority of cells exhibited the dominant pattern of disposition of loci pairs, whereas a small portion cells displayed the reverse pattern. The open bar indicates same cell halves, and the solid bar indicates separate cell halves. Two loci pairs ~180 ° away from each other on the genetic map (1,4; green arrows) exhibited a higher occurrence of dispositions into the same cell halves compared with the pairs ~120 ° away from each other (2,3).

3. Dynamic distribution of chromosomal loci revealed the global conformation reorganization of nucleoid during a cell cycle.

The application of high-resolution fluorescence microscopy techniques and powerful molecular genetics tools provides insight into the dynamic localisation of individual chromosomal loci during cell division cycles. The *oriC* and *dif* loci have unique targeting locations, whereas other loci are distributed linearly between them based on their relative positions in the chromosome map [7, 13]. However, we observed a diversity in gene loci distribution pattern apart from the linear pattern, possibly reflecting the stochastic process of chromosome compaction. For example, as shown in Supplementary Discussion Published Data 3a, the *gln* locus is much closer to the *oriC* locus than to the *lac* locus in the gene map, but the *lac* locus appears nearer to *oriC* than *gln* in the nucleoid according to FISH analysis [12]. This result can be easily understood if they are distributed in a DNA toroidal spool. The relative

positions of loci in newly bilobed sister nucleoid altered dramatically compared with those in newborn cells, indicating a global conformation re-arrangement of the sister nucleoid. A similar phenomenon was observed previously [18] (Supplementary Discussion Published Data 3b). These observations could possibly reflect the transition of nucleoid conformation during cell division to allow the locus to dramatically change its location.

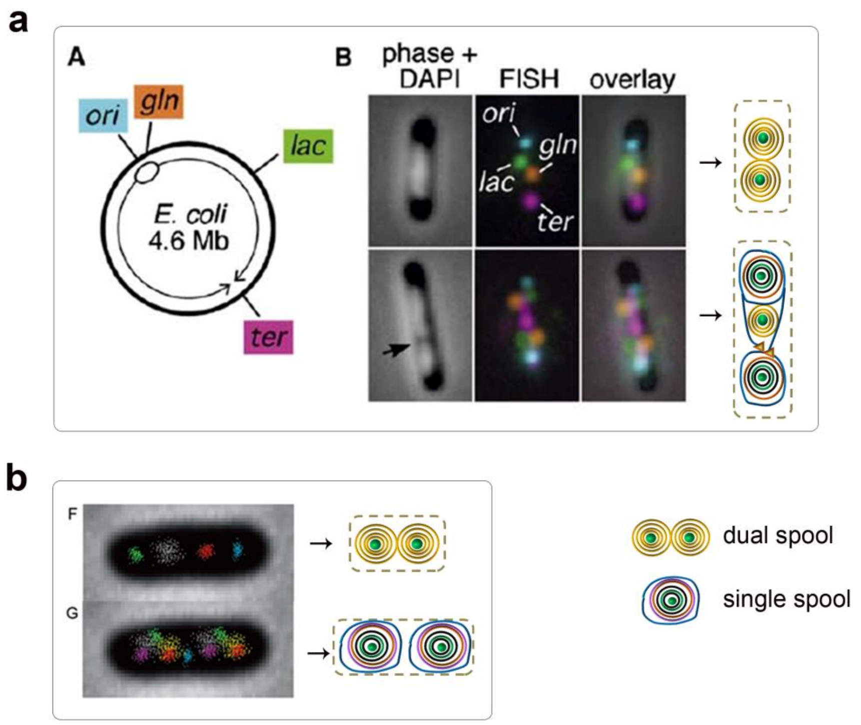

Supplementary Discussion Published Data 3. Localisation of chromosomal loci in newborn cells differs from that in middle-aged cells [12, 18]. (a, b) Fluorescent chromosomal loci in newborn cells (upper panels) were distributed in a pattern different from that in middle-aged cells (lower panels). The diagram indicating the corresponding conformation of chromosome DNA is shown on the right panels.

4. Asymmetry of newly bilobed sister nucleoid.

An unexpected feature of chromosome organization in *E. coli* is the asymmetry of newly split sister nucleoids. When bilobed nucleoids initially appear, they are asymmetric at around the late stage of DNA replication [10, 12] (Supplementary Discussion Published Data 4). The bilobed nucleoids are shaped like dumbbells with a prominent central connection. The two lobes then become equal in size, and they are disposed symmetrically in the cytoplasm with no central connection when DNA replication is complete. This finding agrees well with the model we proposed that one of the docked spools transferred across Ter site to locate in other side of the cell (T3 to T4 transition, Supplementary Discussion Diagram 1b) during the late stage of DNA

replication. Strikingly, the travel of DNA spool acting as a solid explains the ‘dramatic one co-ordination pulsed transition’ as described by the authors [10, 12]. This translocation leaves significant extra room at the original side for the sub-sized sister nucleoid, which might make it clearly visible by DAPI staining. Meanwhile, the prominent central connection could be very possibly the newly synthesised DNA strands from un-replicated parental DNA to be the entering and exiting ends of each sister DNA spool. Due to molecular crowding and confinement of bacteria, it has been difficult to observe directly the dynamic organization of nucleoid in vivo. Previous remarkable findings on nucleoid behaviours in vivo provided precious clues for understanding and determining a possible underlying mechanism that orchestrates the organization and replication of bacterial nucleoids.

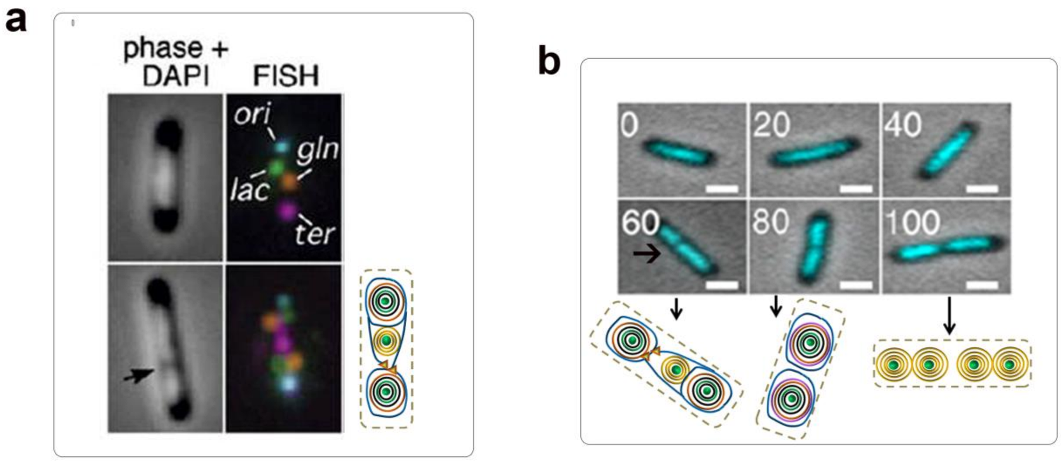

Supplementary Discussion Published Data 4. Asymmetry of new splitting sister nucleoid in *E. coli* [10, 12]. (a, b) Newly split sister nucleoids in a configuration drawn in the bottom right panel in (a) and bottom left panel in (b). One of the thus-far-replicated sister nucleoids at midcell translocated across the non-replicated parental DNA to the other side of cells, resulting in the asymmetry of newly split nucleoids.

5. Development of HNS clusters in nucleoids in vivo.

HNS is a global gene silencer and a main structural protein in the organization of bacterial chromosome [22]. HNS has two modes to shape DNA, namely, the stiffen mode and bridging mode [23]. The stiffen mode is related to gene regulation, and the bridging mode directly modulates DNA condensation. Recently, single molecular studies have revealed the detailed behavioural characteristics of HNS. Divalent ions can switch the transition between two modes [23], and a high off-rate of HNS bridging mode ensures the condensation of DNA but leaves DNA accessible for motor

proteins [24]. With the application of super-resolution PALM, the remarkable features of HNS clusters in live *E. coli* have been revealed, which are distinct from other NAPs dispersed throughout nucleoids [25]. Wang et al. found two HNS clusters in newborn cells, and three to four HNS clusters in growing and ready-to-divide cells (Supplementary Discussion Published Data 5a). One copy of chromosome DNA bears two HNS clusters symmetrically distributed along the long axis of nucleoids (¼ and ¾ positions). This phenomenon is reminiscent of the auto-packaging behaviour of plasmid DNA, resulting in a dual-toroidal-spool conformation with the highest DNA density at the centre core of spools (also ¼ and ¾ positions). The HNS bridging mode depends significantly on the proximity of DNA strands [24]. Thus, HNS is presumed to reside in the centre core of DNA spools because of the close proximity of DNA strands in this area, whereas HNS are not accessible to form significant DNA bridges in the outer area with relatively loosely condensed DNA. HNS serves as a spacer and also stabilizer chaperone of nucleoid in our proposed model. This role agrees with the experimental findings, i.e., the deletion of HNS gene has only a small effect on the growth rate of bacteria strain [26, 27]; nucleoids purified from the HNS-null strain expand approximately five fold more than that observed in WT bacteria [9].

The dynamic development tendency for HNS clusters was schemed in Supplementary Discussion Published Data 5b. How can two clusters develop into three? How can three clusters develop into four to be ready for division? The dynamic pathways were explained by our proposed model for chromosomal DNA organization, and verified by in vivo observation of HNS clusters using PALM. We would like to propose several factors involved in the organization of nucleoids in *E. coli* based on our observations and related knowledge. First, the supercoiled circular DNA itself underwent turbulent fluctuation of conformation upon injection of bending and unwinding mechanical stress by NAPs, and the stress could not be released from the ends of DNA as easily as linear DNA. Instead, the stress propagated along the circular DNA backbone. Stressed nucleoids gradually settled down into a self-balanced conformation to obtain the lowest energetic state. Second, when DNA replication was initiated, the DNA strands were pulled to be processed through the replication factory in *E. coli* [28, 29]. In this course, locally bound chaperone NAPs are likely to dissociate by the pulling force generated (~30 pN), and the torque stress on this portion of DNA is relieved. Concomitantly, topological insulators of nucleoids ensure that the local pulling and torque relieving effects do not travel far to trigger global topological conformation alteration [30, 31]. Third, NAPs such as HNS, HU, Fis and IHF ect., are not the governor to shape and hold tightly a compact nucleoid, instead, they are dynamically bound and dissociated from nucleoid and the overall torque effect introduce DNA to transit from free diffusion state into packaged state. However, the fundamental mechanism for auto-organization of highly ordered chromosome DNA in cells is waiting to be discovered as the proceedings of single molecular studies and dynamic in vivo investigations in future.

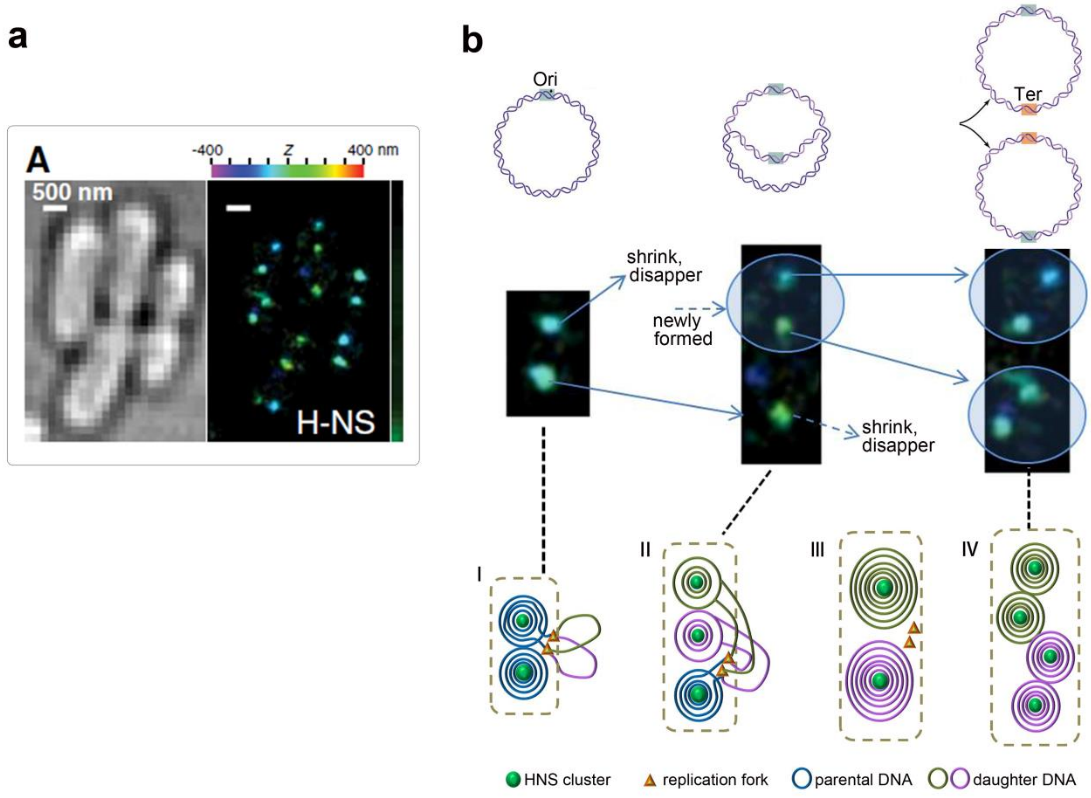

Supplementary Discussion Published Data 5. HNS cluster distribution in *E. coli* cells [25]. (a) Two HNS clusters in newborn cells, three HNS clusters in growing cells and four HNS clusters in ready-to divide cells were previously observed in *E. coli*. **(b)** The dynamic developing tendency of HNS clusters was schemed in middle panel. Upper panel, DNA replication stage. Bottom panel, chromosome DNA conformation during a cell cycle. I, early stage of DNA replication; II, middle stage of DNA replication; III, complete of DNA replication; and IV, pre-division stage.

6. Dynamic observation of morphology of nucleoids in live *E. coli.*

The dynamic morphology of nucleoids has been extensively studied in recent years [8, 17, 32-35]. New microscopic techniques and labelling strategies have revolutionised our opinions of nucleoids, as revealed by super temporal and spatial resolution microscopy. The striking features of nucleoids are addressed and discussed in this section.

4D imaging of *E. coli* nucleoids with mCherry-tagged HU protein, a dynamic helical ellipsoid was presented [33] (Supplementary Discussion Published Data 6a). The discrete, dynamic ellipsoids within a nucleoid were typically found in growing *E. coli* cells. This observation was consistent with our model; the fluctuant developing DNA spools are reminiscent of dynamic ellipsoids. DAPI staining of *E. coli* cells growing in Lennox rich medium revealed two to four blocks of DNA in cells [17] (Supplementary Discussion Published Data 6b). In such growth conditions, DNA

replication proceeded without an isolation state from replication forks. Therefore, single-spool could be the favoured packaging conformation for DNA mass. The DNA blocks display similar morphology as DNA spools.

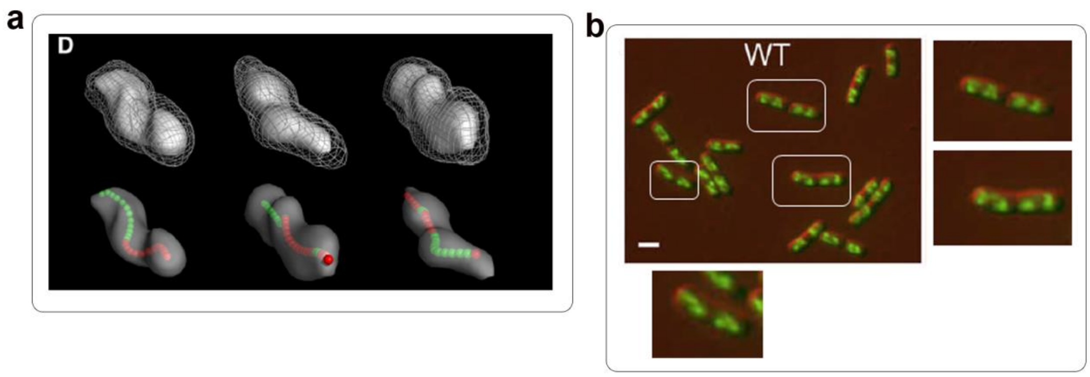

Supplementary Discussion Published Data 6. Morphology of nucleoids in *E. coli* cells. (a) 4D imaging of mCherry-tagged HU revealed dynamic ellipsoid structures of nucleoids [33]. **(b)** DAPI staining of nucleoids in MG1655 strain in Lennox rich medium, showing two to four blocks of chromosome DNA in cells [17]. The enlarged images of the regions enclosed in white-line-boxes are listed at right and bottom panels.

(Notably, numerous DAPI-stained nucleoids in live *E. coli* cells under slow growth conditions demonstrate linear fluorescent morphology, as shown in Supplementary Discussion Published Data 4b. The difference between linear and blocks appearance possibly derives from growth conditions. Under fast growing condition, nucleoid is more compacted than in the slow growing media, which may be due to faster synthesis rate of NAPs than that in cells growing in slow growing media. The more compacted, thus more isolated nucleoid, may allow more obvious visualisation of transition of DNA global conformations than loosely compacted nucleoid where fluorescent DNA blocks were not separated enough and blurred with each other.)

Using an inducible GFP-tagged Fis protein to image the entire chromosome, the growth and division of nucleoids during multifork replication was observed in cells growing in grooves under LB-agarose pad [8] (Supplementary Discussion Published Data 7a). One block of chromosome DNA in pre-division cells became bilobed in newborn cells, and the two lobes were separated and isolated in the next cell cycle. The organization and segregation patterns of the multifork *E. coli* chromosome are schemed in Supplementary Discussion Published Data 7b and c. In brief, chromosome DNA is organized into single-spool docking on replication forks before the completion of DNA replication. Given that the initiation of a new round of DNA replication occurs earlier than the end of whole chromosome replication, the newly synthesised sister DNA is stressed by NAPs binding and collapses into two new single-spools before the disassembly of the parental spool. Sister spools grow and the parental spool shrinks as multiple fork replication proceeds. The parental spool finally turns into a central connection and disappears. Thus, the sister nucleoids are isolated from one another.

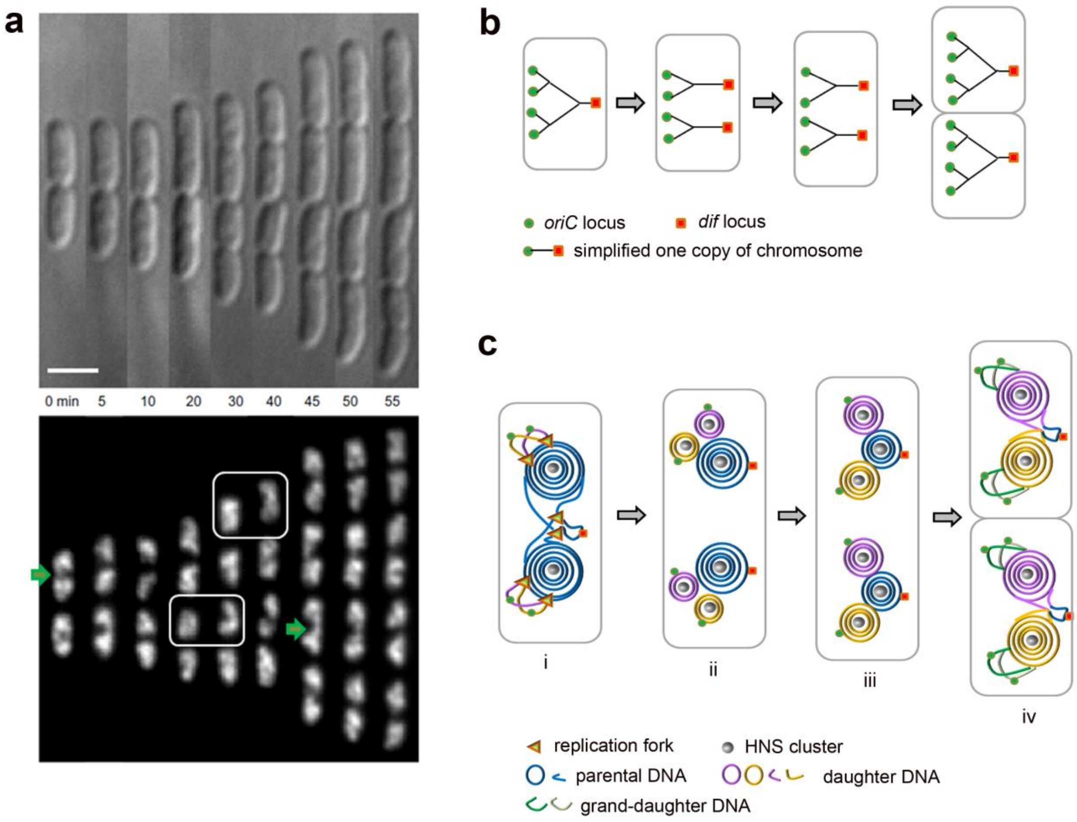

Supplementary Discussion Published Data 7. Morphology of multifork *E. coli* chromosome during cell division cycles [8]. (a) The GFP-tagged Fis protein allows visualisation of nucleoids during a cell cycle. **(b)** Diagram of a cycle of multiple-fork replication of chromosome DNA. **(c)** Proposed DNA organizational pattern of nucleoids in different ages during a cell cycle corresponding to (b). Conformations ii and iii explain the bilobed transition of nucleoids observed experimentally (white line boxes, in a). i and iv indicate nucleoids with central connection (green arrows, in a). Replication forks were omitted in ii–iv.

Helical bundles of nucleoids in *E. coli* have been proposed by both experimental and theoretical investigations [7, 8, 33]. In direct scanning of nucleoids by monitoring GFP-tagged Fis protein or mCherry-tagged HU protein, significant helical bundles were observed using image analysis by deconvolution [8, 33] (Supplementary Discussion Published Data 8a,b). In these observations, right-handed and left-handed helicity were found even along the length of a single nucleoid, indicating no particular handedness in nucleoids. In our 3D model, the DNA strands in dual-toroidal-spool rotated along their orbits to compensate for the bending tension stress, as proposed by theoretical analysis [1]. Thus, global helicity was introduced into the nucleoid by this rotation. The upside down relationship between the two spools sharing similar configuration (Supplementary Fig. S7) resulted in right-handedness in one spool and left handedness in the other spool as observing the nucleoid from same perspective, thereby eliminating the overall handedness of DNA bundles in a nucleoid

(Supplementary Discussion Published Data 8d). Another feature of our model for nucleoids is that DNA density increased from the periphery to the centre core of the spool. The bending energy was significantly higher in the centre core than that in the periphery (Supplementary Fig. S5). HU and Fis proteins were sensitive to tension; the association rate significantly decreased when the tension increased [36, 37]. Thus, the centre core of mature DNA spools of nucleoids was dominated by non-bending NAPs, such as HNS in bridging mode, which was favoured by closely juxtaposing DNA in the centre core. Other DNA bending proteins had difficulty in conquering the bending energy in DNA located in the centre core, so they were mainly abundant at the periphery of spools. The periphery DNA bundle morphology derived from dual-toroidal-spool conformation of nucleoids is schematically shown in Supplementary Discussion Published Data 8 e and f, which was in agreement with the observations documented.

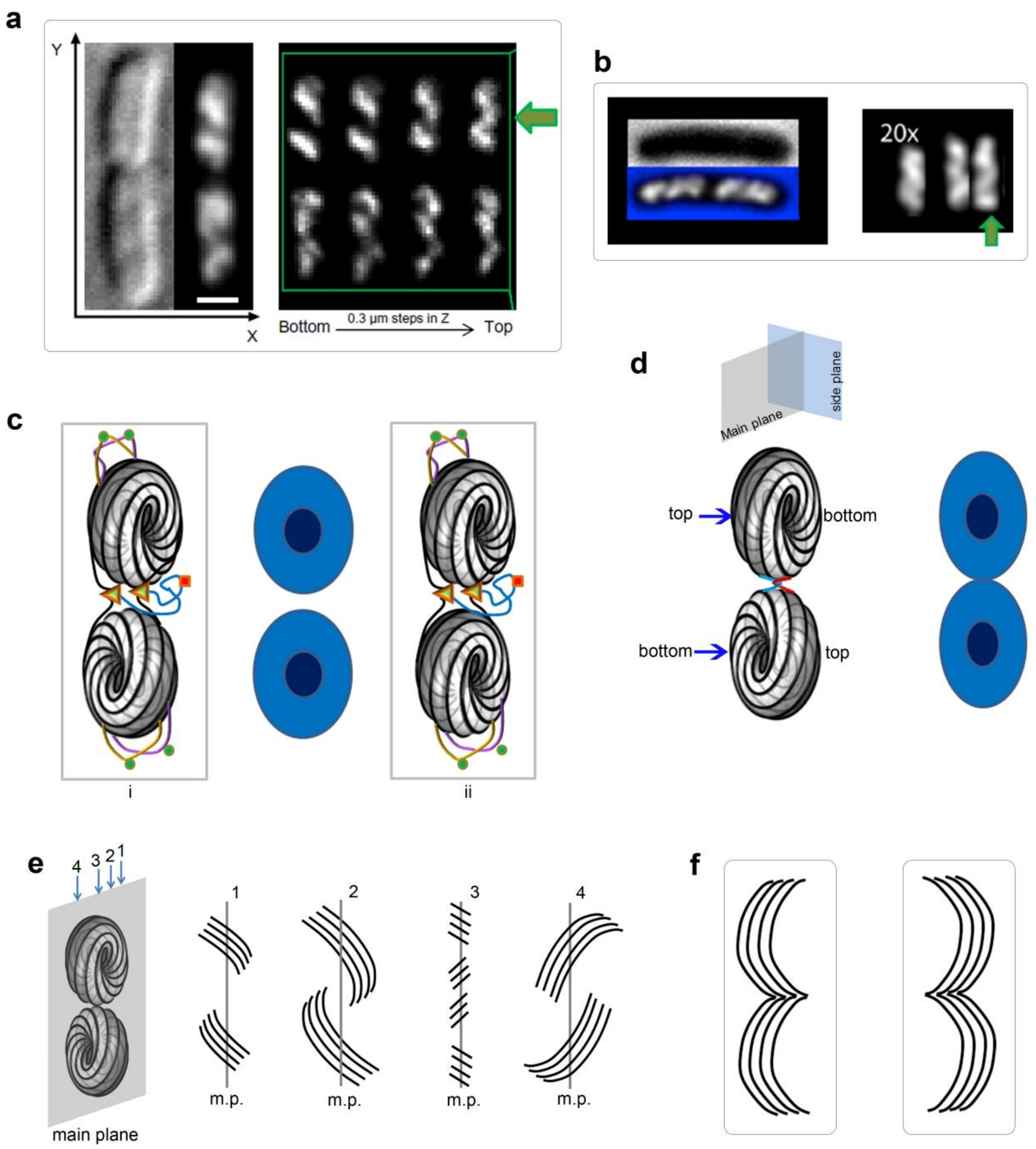

Supplementary Discussion Published Data 8. Helical bundles of nucleoids. (a, b) The nucleoid was scanned by fluorescent Fis or HU proteins, and helical bundles of the nucleoid were revealed previously [8, 33]. **(c)** DNA conformations proposed for newborn cells (arrow, a) with multifork replication of chromosome DNA (i, ii). Green spot, *oriC* locus; red square, *dif* locus; triangle, replication fork. The binding profile of HU or Fis is shown in the middle panel. The centre core (dark blue) was void of significant HU or Fis protein binding because of high bending energy, whereas the periphery of the nucleoid (light blue) was bound by abundant HU and Fis proteins. **(d)** DNA conformation for newborn nucleoids in slow growth conditions (arrow, b) is shown in the left panel. The helical bundles of nucleoids originate from the 3D rotation of DNA strands along their orbits, as elucidated by the 3D model of dual-toroidal-spool conformation. The upside down relationship eliminated the particular handedness inside a nucleoid. The protein binding profile is in the right panel. **(e)** Sections of nucleoids along the main plane (blue arrows, left panel) are shown on the right. The DNA strands on each section are drawn by black lines. m.p. indicates main plane as indicated in (d). **(f)** At lateral view from both sides of nucleoid**,** the overall curvature of a dual-spool was simplified shown in the boxes. Each spool contributes a “C” shape curvature. The “two-tandem-C” shape resembles the curvature of nucleoids in (a) and (b).

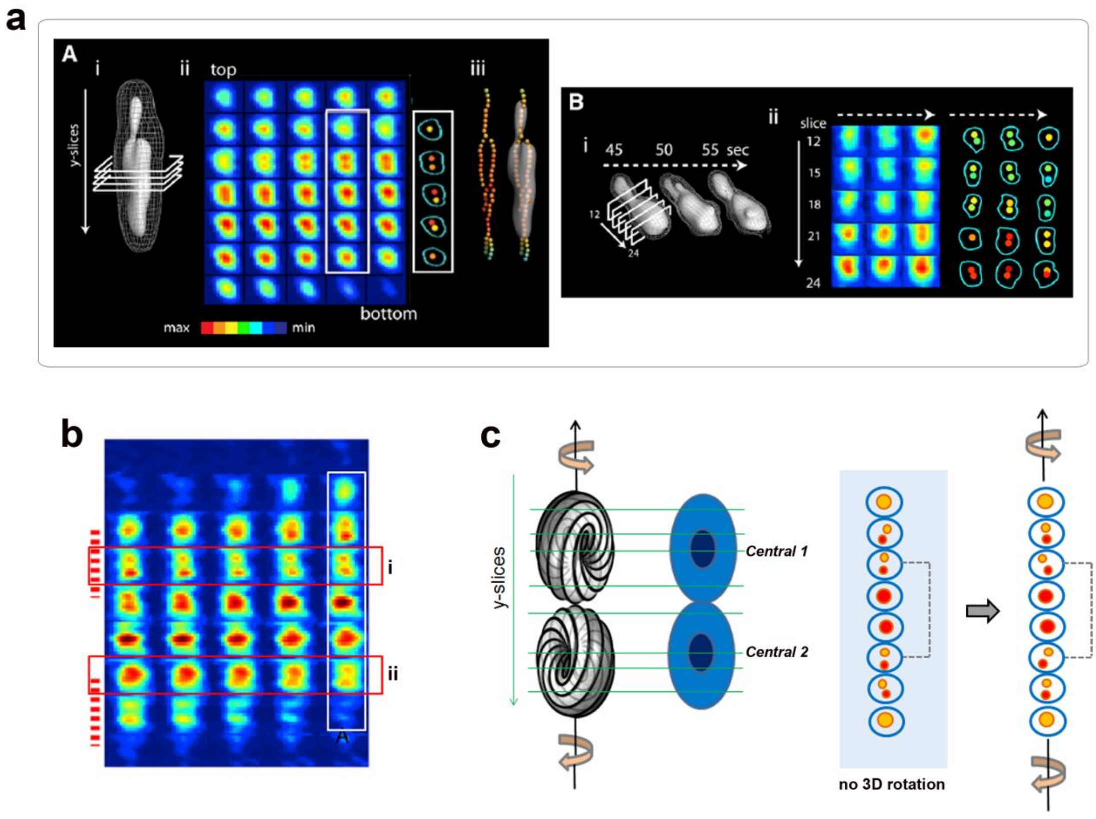

Supplementary Discussion Published Data 9. Distribution of fluorescent HU proteins along the long axis of the nucleoid. (a) Distribution of fluorescent HU protein along the y-slices shows a two-helical-bundle pattern, as presented previously (iii, left panel) [33]. The dynamic fluctuation of fluorescent signals was significant on the 5 s scale, as shown in the right panel. Fluctuation was probably due to the association–dissociation dynamics of HU proteins to DNA, as well as turbulent fluctuation of DNA strands in nucleoids. **(b)** The images of nucleoids during the early stage of DNA replication. At this

time point (before T1, as mentioned by the authors), one spool in dual-spool conformation already replicated and slightly shrank, based on the lack of fluorescence in the lower panels (panels below ii). This image was selected as a representative for comparison with a mature dual-toroidal-spool because ~62±14% newborn cells underwent dynamic fluctuation of their conformation with a sub-matured un-balanced distribution of two HNS clusters, as observed in our experiments. DNA replication was often observed after the appearance of two symmetrically comparable HNS clusters. Thus, the nucleoid in early DNA replication stage was considered to retain, at least partially, the balanced mature dual-toroidal-spool conformation with aids of topological insulators in *E. coli*. **(c)** Corresponding y-slices of nucleoid based on our model. The green lines in the left panel indicate the positions of arbitrary slices. Given that the centre core of each spool (dark blue) prevented significant binding of HU protein, the periphery of the spool was bound with abundant HU proteins. Therefore, the fluorescence signals from the top to bottom exhibited a "one-two-one-two-one" pattern based on our model (right panel), which is substantially same as the pattern shown in (**b**, white line box). Due to the counterclockwise rotational tendency of the spool along the long axis of nucleoid to the hinge, the two spools rotated and finally buckled together to form a small dihedral angle between the two spools (dashed line, right panel), in comparison with the case without rotational tendency of DNA spools to the hinge (dashed line, middle panel). Coincidently, experimental observations display a dihedral angle between the two planes (the planes across two bundles, red-line-box i and ii in **b**).

As recently reported, nucleoids in live *E. coli* exhibit striking features in temporal and spatial organizations [33]. The authors performed 4D scanning of nucleoids using mCherry-tagged HU protein. They observed 'dual longitudinal helical bundles' of nucleoids. Surprisingly, the observations fit well with our model. As shown in Supplementary Discussion Published Data 9, nucleoids illuminated by fluorescent HU protein largely displayed a 'one-two-one-two-one' pattern of HU-tagged bundles from one pole to the other pole. We explain this feature with our proposed model as follows. The centre core lacked HU proteins, but HU was abundant at the periphery. Thus, two bundles were observed in the middle part of the spool, but one bundle was detected in the distal ends of the spool (Supplementary Discussion Published Data 9c). When the nucleoids were in the process of replication, specifically, in the late phase of DNA replication (Supplementary Discussion Published Data 10), the newly formed docked spool or isolated spool underwent turbulent fluctuation. The conformation was not balanced yet, so we presumed that tension in the centre core of such nucleoids was not high enough to avoid significant HU binding. At these time points, HU proteins exhibited one large alternating bundle with a fluorescent density slightly higher than that of nucleoids in newborn cells.

There is a counterclockwise 3D rotational tendency of the spool along the long axis of the nucleoid to the hinge, as suggested by our model (Supplementary Fig. S8). Coincidently, the experimental observation demonstrated a small angle between the two lines passing through both bundle signals in one slice (between i and ii, Supplementary Discussion Published Data 9b). The slices of periphery DNA bundle distribution in dual-toroidal-spool conformation are schematically shown in Supplementary Discussion Published Data 9c, which were in agreement with the experimental results.

It is striking that the pulsed nucleoid growth in length at defined time points during a cell cycle was revealed in their study [33] (Supplementary Discussion Published Data 11). The first pulse was observed when DNA replication was halfway finished, which coincided with the collapse of newly synthesised DNA into two docked spools in our model. At this time point, the authors mentioned the growth direction towards the old pole of cells, which was in agreement with our observation that two additional spools were initially distributed at the side of the old pole. There is the second pulse of growth of nucleoid toward the other cell pole. We attributed this second pulse to the translocation of one docked spool from the centre to across the remaining parental spool during the late age of DNA replication. In another study, sister chromosomes were described to lose cohesion and separate in a single coordinate transition involving most or all of the thus-far-replicated regions at late stage of DNA replication [12]; these findings were consistent with our observations by PALM. The latter pulse growth of nucleoids was explained by our model as follows. At third pulse, DNA replication was completed and the docked new spools were isolated from the replication factory to move freely to fit into their respective cell halves. At fourth pulse, two isolated single-spools rearranged into dual-toroidal-spool conformation, which slightly elongated the length of nucleoids for geometric reasons. To our surprise, our model presenting the dynamic organization of chromosomes in *E coli* was in good agreement with the direct experimental observations.

Due to limitation of manuscript, we cannot discuss many other interesting published data about bacterial chromosome organization. Overall, in order to probe the complicated question of chromosome DNA organization in *E. coli*, a new perspective in combination of physical deduction and biological integration was regarded in the present study for understanding of the underlying mechanisms. It has been extremely difficult to directly monitor DNA compaction in a crowded tiny *E. coli* cell. However, considering that chemical and physical principles govern every biochemical reaction and behaviour of single molecules, and also considering that the complicated and subtle experimental signs from in vivo observations are the integrated reflections of cytoplasmic events, we checked the model deduced from single molecular observations with thus-far-discovered in vivo behaviours of nucleoids from the literature. Our proposed model accommodated majority of the remarkable features of nucleoids in *E. coli* previously observed in vivo. The DNA itself plays a major role in its organization, as shown in the present study. NAPs are the auxiliary factors to deform local DNA strands, so the accumulation of the local torque effect induced a global transition of DNA conformation from free diffusion into a packaging spool, which was the major opinion of this study. The findings provide new perspectives to understand chromosome organization in *E. coli*. The underlying physical principles may expand our understanding of the auto-organization behaviours of DNA in other bacteria and eukaryotes.

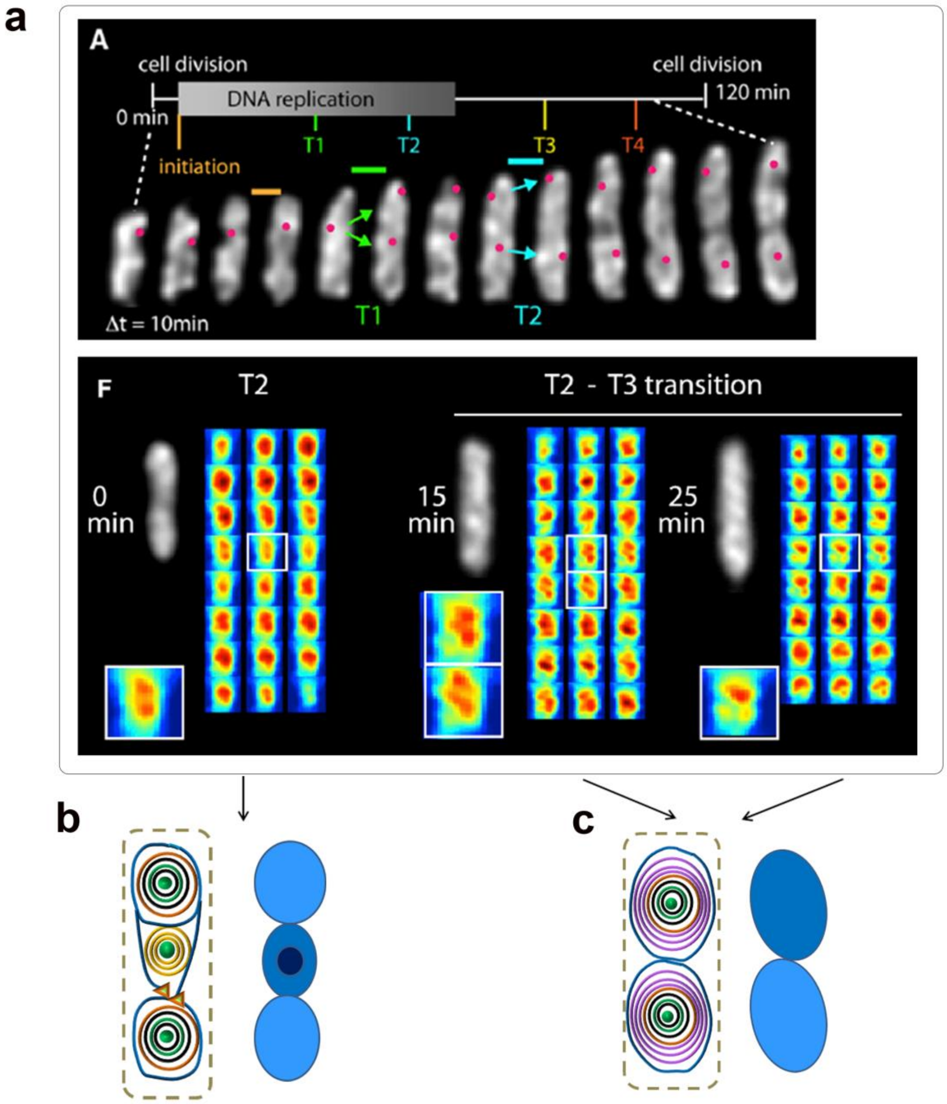

Supplementary Discussion Published Data 10. Fluorescent HU bundles in replicating nucleoids (a) [33]. We would like to explain the T2 bundles with the diagram in **(b)**. The newly formed DNA spools located at upper and bottom panels, are abundantly bound with HU at this time span when a spool is still immature. Whereas in the middle panel, the parental spool retains partially its original mature conformation during replication, thus the centre core lacking HU binding resulted in two bundles of HU. We would like to attribute the irregular fluorescent bundles to “newly-developing-spool” as shown in **(c),** wherein the turbulent fluctuation of the spools and prosperously HU binding may thus result in multi-bundles of HU proteins in irregular shape along the nucleoid.

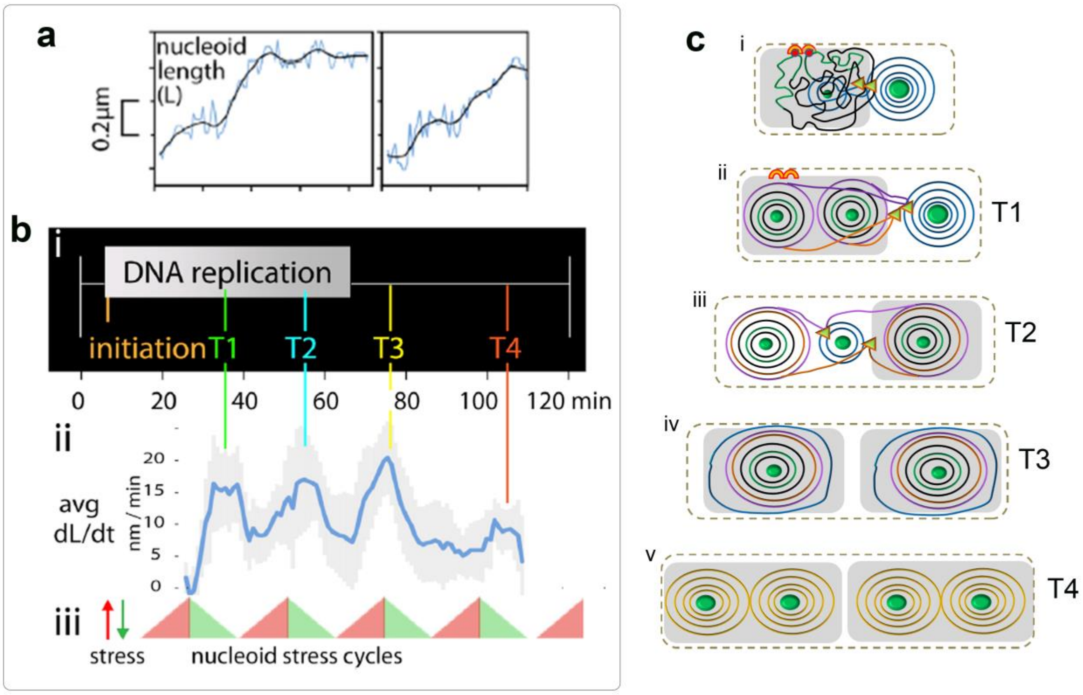

Supplementary Discussion Published Data 11. Discontinuous sequential elongation pulses of nucleoid at defined times in the cell cycle [33]. (a-b) Four discontinuous pulses in the growing of the length of nucleoid, at T1 to T4 in the cell cycle (b). Meanwhile, a short period of nucleoid shortening was detected following the period of length increase (a). **(c)** The corresponding increase in length of nucleoid at T1 to T4 was highlighted in ii to v, as suggested by our model. The events are briefly described as follows: at T1, freely defusing DNA collapsed into two additional spools; at T2, one of the new spools translocated to the other side of cells; at T3, the docked new spools isolated from the replication factory and moved into their cell halves; at T4, each spool containing one sister chromosome rearranged into the final balanced dual-toroidal-spool conformation. We speculate that the short period of nucleoid length shortening as shown in (a) may possibly due to the continuous compaction of the spools thus make it more tightly and shrink a bit transiently, or the central connection pull the spools back a bit elastically in response to the sudden movements arising from collapse into spools (T1) or translocation (T2).

Supplementary methods

Atomic force microscopy

The plasmid DNA pBR322 was platinated and subsequently imaged by AFM as described previously [38]. pBR322 DNA at 5 ng/μl was incubated with C (r_i = 0.4, r_i is the molar ratio of platinum complex to nucleotide), or with a C–T mixture (at 1:1 molar ratio) in 10 mM $NaClO_4$ at 37 ℃ for 5 h in the dark. At 1, 2, 3, 4, and 5 h of incubation, one portion (~2 μl) of the sample was subjected to AFM imaging. The reaction was stopped in the remaining portions of samples, which were then purified and subjected to inductively coupled plasma mass spectrometry (ICP–MS) test to measure the bound platinum amount per DNA molecule. Samples of >5 h incubation were excluded from analysis because of the intense condensation of plasmid DNA molecules by platination, which is beyond the scope of this study. The sharp bends per DNA molecule were calculated according to the relationship that the amount of sharp bends equals 16% of the total divalent bound platinum amount per DNA molecule, as determined by ICP–MS and denaturing gel electrophoresis analyses [38]. The writhe number was checkd manually using the acompanying software of AFM. The results of writhe number were confirmed by measuring the height value of the points where DNA strands crossed over with each other. In such joints, the height value was about twice or more of that of a dsDNA strand.

Construction mEos2-tagged HNS endogenous strain

The parent strain was MG1655 [39]. The chromosomal fusion of mEos2 and HNS gene was constructed using λ Red recombination [25, 40]. MG1655 bacterial strain-carrying pKD46 plasmids was electronically competent, and transformed with 100–500 ng of linear functional cassette DNA flanked with homologous sequences of HNS by an electroporation machine (Bio-Rad, USA). The mEos2 expression plasmids were purchased from Addgene (USA) [41]. The FRT flanked chloramphenicol resistance gene (CAM) selection cassette was purchased (Novagene, Germany). The mEos2 and CAM selection cassette was ligated by NotI restriction enzyme site. The functional cassette for HNS-mEos2 fusion construction was prepared by PCR, amplifying the mEos2-FRT-Chlora-FRT fragments with primers containing the ~56 nt homologous sequences flanking the HNS gene. Successful recombination was first selected by CAM antibiotic resistance marker, followed by colony PCR screening and gene loci sequencing. The primers used are listed below.

H1-P1-HNS-mEos2,
CAAAAAAGCAATGGATGAGCAAGGTAAATCCCTCGACGATTTCCTGATCAAGCAAATGAGTGCGATTAAGCCAG (55nt homology extensions)

H2-P2-HNS-mEos2,
GGACAATAAAAAATCCCGCCGCTGGCGGGATTTTAAGCAAGTGCAATCTACAAAAGATAATACGACTCACTATAGGG (57nt homology extensions)

Sample preparation for imaging

For imaging, the colony containing successful fusion constructs was selected and incubated in 3 ml of LB media for 5 h. The cells were diluted at 1:10,000 into M9 minimal media supplied with vitamin and amino acids (Invitrogen, USA) or LB media, and cultured at 250 rpm and 37 ℃ overnight. The cells were dilluted at 1:1000–1:2000 into fresh M9 media (or LB media) and cultured at the same conditions for 2–4 h to reach an OD_{600} of ~0.1 the next morning. Approximately 200 μl of the culture was disposed onto a Petri dish precoated with 0.3% poly-L-lysine as described previously [42]. The cells were allowed to immobilise onto the Petri dish for 30–45 min before imaging, and maintained at room temperature. Subsequently, 3 ml of fresh M9 complete media (or LB-agarose pad) was added into the Petri dish, and the cells were subjected to imaging.

Imaging and data analysis

A fluorescent microscope and EMCCD were used as described previously [43]. The associated items (e.g., oil immersion objective, laser, mechanical shutter) were purchased and assembled as described prevously [25]. The excitation and emission paths were established as previously performed [25]. In our study, one to multiple cell cycles were monitored by fluorescent HNS-mEos2 clusters. Extensive emmission at 405 nm would harm cellular DNA and other cytoplamic components. Cells need 10–15 min to recover from single PALM imaging to continue to grow [28]. To reduce phototoxicity by illumination at 405 nm, time lapse snapshots were obtained at time intervals of 15, 20, and 25 min. 2−4 decades of mEos2 fluorophore per cell at each time was photoactivated by the 405 nm excitation (30−60 W cm^{-2}) over the duration of 100−500 ms. Two to three snapshots were taken under 20 W cm^{-2} 561 nm excitation at 150 ms per frame. Subsequently, the fluorophore was photobleached. Bright field images of the cell contours were acquired before PALM imaging. The original images from ~700 cells was processed by image J software and sequentially analysed based on the time during a cell cycle. The data were integrated to analyse the reprensentive phase information of HNS cluster developments during the cell division cycle.

Supplementary Reference

[1] I.M. Kulic, D. Andrienko, M. Deserno, Europhys Lett 67 (2004) 418-424.

[2] A. Rich, S. Zhang, Nat Rev Genet 4 (2003) 566-572.

[3] I.F. Lau, S.R. Filipe, B. Soballe, O.A. Okstad, F.X. Barre, D.J. Sherratt, Mol Microbiol 49 (2003) 731-743.

[4] M. Thanbichler, P.H. Viollier, L. Shapiro, Curr Opin Genet Dev 15 (2005) 153-162.

[5] R. Reyes-Lamothe, X. Wang, D. Sherratt, Trends Microbiol 16 (2008) 238-245.

[6] J. Pelletier, K. Halvorsen, B.Y. Ha, R. Paparcone, S.J. Sandler, C.L. Woldringh, W.P. Wong, S. Jun, Proc Natl Acad Sci U S A 109 (2012) E2649-2656.

[7] P.A. Wiggins, K.C. Cheveralls, J.S. Martin, R. Lintner, J. Kondev, Proc Natl Acad Sci U S A 107 (2010) 4991-4995.

[8] N. Hadizadeh Yazdi, C.C. Guet, R.C. Johnson, J.F. Marko, Mol Microbiol 86 (2012) 1318-1333.

[9] V.V. Thacker, K. Bromek, B. Meijer, J. Kotar, B. Sclavi, M.C. Lagomarsino, U.F. Keyser, P. Cicuta, Integr Biol (Camb) 6 (2014) 184-191.

[10] M.C. Joshi, A. Bourniquel, J. Fisher, B.T. Ho, D. Magnan, N. Kleckner, D. Bates, Proc Natl Acad Sci U S A 108 (2011) 2765-2770.

[11] E. Toro, L. Shapiro, Cold Spring Harb Perspect Biol 2 (2010) a000349.

[12] D. Bates, N. Kleckner, Cell 121 (2005) 899-911.

[13] H. Niki, Y. Yamaichi, S. Hiraga, Genes Dev 14 (2000) 212-223.

[14] M. Valens, S. Penaud, M. Rossignol, F. Cornet, F. Boccard, EMBO J 23 (2004) 4330-4341.

[15] Y. Yamaichi, H. Niki, EMBO J 23 (2004) 221-233.

[16] O. Danilova, R. Reyes-Lamothe, M. Pinskaya, D. Sherratt, C. Possoz, Mol Microbiol 65 (2007) 1485-1492.

[17] R. Mercier, M.A. Petit, S. Schbath, S. Robin, M. El Karoui, F. Boccard, O. Espeli, Cell 135 (2008) 475-485.

[18] O. Espeli, R. Mercier, F. Boccard, Mol Microbiol 68 (2008) 1418-1427.

[19] H.J. Nielsen, J.R. Ottesen, B. Youngren, S.J. Austin, F.G. Hansen, Mol Microbiol 62 (2006) 331-338.

[20] X. Wang, X. Liu, C. Possoz, D.J. Sherratt, Genes Dev 20 (2006) 1727-1731.

[21] A. Thiel, M. Valens, I. Vallet-Gely, O. Espeli, F. Boccard, PLoS Genet 8 (2012) e1002672.

[22] C.J. Dorman, Nat Rev Microbiol 2 (2004) 391-400.

[23] Y. Liu, H. Chen, L.J. Kenney, J. Yan, Genes Dev 24 (2010) 339-344.

[24] R.T. Dame, M.C. Noom, G.J. Wuite, Nature 444 (2006) 387-390.

[25] W. Wang, G.W. Li, C. Chen, X.S. Xie, X. Zhuang, Science 333 (2011) 1445-1449.

[26] T. Atlung, F.G. Hansen, J Bacteriol 184 (2002) 1843-1850.

[27] R. Srinivasan, D. Chandraprakash, R. Krishnamurthi, P. Singh, V.F. Scolari, S. Krishna, A.S. Seshasayee, Mol Biosyst 9 (2013) 2021-2033.

[28] S. Uphoff, R. Reyes-Lamothe, F. Garza de Leon, D.J. Sherratt, A.N. Kapanidis, Proc Natl Acad Sci U S A 110 (2013) 8063-8068.

[29] R. Reyes-Lamothe, D.J. Sherratt, M.C. Leake, Science 328 (2010) 498-501.

[30] L. Moulin, A.R. Rahmouni, F. Boccard, Mol Microbiol 55 (2005) 601-610.

[31] L. Postow, C.D. Hardy, J. Arsuaga, N.R. Cozzarelli, Genes Dev 18 (2004) 1766-1779.

[32] S. Adachi, T. Fukushima, S. Hiraga, Genes Cells 13 (2008) 181-197.

[33] J.K. Fisher, A. Bourniquel, G. Witz, B. Weiner, M. Prentiss, N. Kleckner, Cell 153 (2013) 882-895.

[34] B. Youngren, H.J. Nielsen, S. Jun, S. Austin, Genes Dev 28 (2014) 71-84.

[35] G.S. Gordon, D. Sitnikov, C.D. Webb, A. Teleman, A. Straight, R. Losick, A.W. Murray, A. Wright, Cell 90 (1997) 1113-1121.

[36] B. Xiao, H. Zhang, R.C. Johnson, J.F. Marko, Nucleic Acids Res 39 (2011) 5568-5577.

[37] B. Xiao, R.C. Johnson, J.F. Marko, Nucleic Acids Res 38 (2010) 6176-6185.

[38] Y.R. Liu, C. Ji, H.Y. Zhang, S.X. Dou, P. Xie, W.C. Wang, P.Y. Wang, Archives of Biochemistry and Biophysics 536 (2013) 12-24.

[39] Y. Hao, Z.J. Zhang, D.W. Erickson, M. Huang, Y. Huang, J. Li, T. Hwa, H. Shi, Proc Natl Acad Sci U S A 108 (2011) 12473-12478.

[40] K.A. Datsenko, B.L. Wanner, Proc Natl Acad Sci U S A 97 (2000) 6640-6645.

[41] S.A. McKinney, C.S. Murphy, K.L. Hazelwood, M.W. Davidson, L.L. Looger, Nat Methods 6 (2009) 131-133.

[42] J. Elf, G.W. Li, X.S. Xie, Science 316 (2007) 1191-1194.

[43] H. Li, Z.W. Duan, P. Xie, Y.R. Liu, W.C. Wang, S.X. Dou, P.Y. Wang, PLoS One 7 (2012) e45465.
